# Geographic network effects to engage people in the energy transition: The case of PV in Switzerland

**DOI:** 10.1016/j.heliyon.2023.e17800

**Published:** 2023-06-30

**Authors:** Gloria Serra-Coch, Romano Wyss, Claudia R. Binder

**Affiliations:** a Laboratory for Human Environment Relations in Urban Systems (HERUS), Institute of Environmental Engineering (IIE), School of Architecture, Civil and Environmental Engineering (ENAC), École Polytechnique Fédérale de Lausanne (EPFL), CH-1015 Lausanne, Switzerland; b Wyss Conseil Scientifique, Rue du Bourg 8, CH-1095 Lutry, Switzerland

**Keywords:** Energy transition, Neighbourhood effects, Photovoltaics, Switzerland, Information networks

## Abstract

With the energy transition and CO_2_ emissions reduction as a priority on the international policy agenda, governments worldwide are trying to engage the population in investing in renewable energies. In this paper, we study the role of information access and peer effects in the photovoltaics sector in the case of the Swiss canton of Vaud. Based on a representative survey of the population of two districts, Nyon and Jura-Nord Vaudois, we show that being a homeowner and knowing someone who has installed PV cells in your social group significantly increase the probability of putting up PV cells. A direct neighbourhood effect was found, meaning that if a neighbour has installed a PV cell, the probability of the inhabitants in the two case study regions installing one themselves increases significantly. Our results show that spatial proximity is an important factor in the transmission of information between peers. Besides pure geographical distance, additional aspects such as administrative boundaries, shared language or degree of urbanisation play a role in the way the information network is presented. These insights indicate that professional experts and neighbours are important points of reference in the decision to invest in PV, and that regional networks are key for the active spreading of information on renewable technologies. Thus, we recommend using these connections to actively promote PV.

## Highlights

• People exchange information on PV with others who are socially and spatially close.
• Spatial proximity seems to matter at regional level for these exchanges to happen.
• Home ownership and professional engagement positively influence PV investment.
• There is potential to use neighbourhood connections for the energy transition.
• Existing knowledge exchange networks are key to spread information regarding PV.

## Graphical abstract

**Figure:**
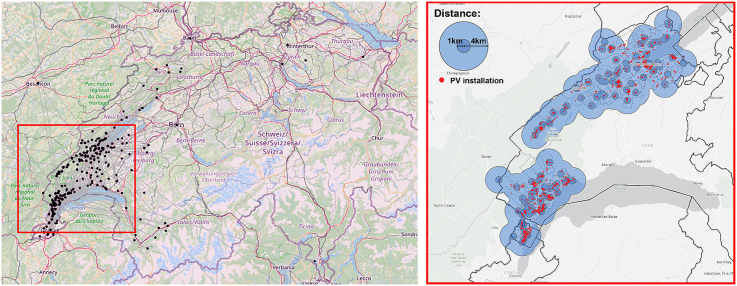

## 1 Introduction

The energy transition is a major challenge for society in all industrialised countries. The expansion of photovoltaic panels (PV) is a key element of energy transitions worldwide [1]. To support the energy transition and the diffusion of PV, actions are required at different levels of government [2,3]. While national and local level have received ample scholarly attention, far less attention has been paid to regional level [4]. However, everyday life decisions are generally taken at regional level, and regional networks have been identified as crucial to foster the energy transition [5].

In parallel to global developments, an increase in photovoltaic energy sources is one of the key pillars of the Energy Strategy 2050 in Switzerland (SFOE, 2020) and has extensive support among the Swiss population [6]. In its direct democratic federal system, the Swiss government and the Swiss people have adopted key policy goals, ranging from phasing out nuclear energy to the accelerated development of renewable energies and the promotion of energy efficiency measures [7]. In Switzerland, the cantons have a major role in the implementation of specific energy-related policies and the coordination of measures that support the national goals by adopting cantonal strategies on energy efficiency, handing out water concessions, financing important subsidy schemes and defining energy standards for the building sector [6]. They are the central political entity at sub-federal level and can be compared in terms of governance power to US American states or German Länder. However, they are relatively small by international standards (between 16,300 and 1,564,000 inhabitants). The district is a regional political entity, which represents an intermediate scale between the municipality and the canton and can be compared to a county in the American context or a Landkreis in Germany.

Regions play an important role in the implementation of the energy transition [8,9]. Regional structures, such as *Energieregionen* in Austria, seem to support the energy transition by acting as contact points and information providers close to the local people [[10], [11], [12]]. This reflects interest in broader issues of culture, norms and learning in specific institutional contexts [13,14]. It also relates to the varying degree of importance given to environmental issues in specific geographic and cultural settings [[15], [16], [17]]. People are more likely to engage in the energy transition if they feel that they are in control of the development and profit either financially or ideologically from the expansion of renewables in their surroundings [18]. However, despite the apparent importance of considering the impact of different geographic scales, several scholars have outlined the lack of attention given to the geographic perspective in the energy transition [[19], [20], [21]]. Thus, it is important to expand our current knowledge within the field of the energy transition adding a geographic lens. In addition, Switzerland is a unique case study to implement this local and geographic perspective, due to the importance of local government, which translates to a fairly diverse sub-national landscape.

Proximity factors also play a central role when it comes to the decision of individual actors to invest in renewable energies in general and photovoltaics (PV) specifically. These factors include geographical, social and psychological proximity [22]. Curtius et al. [23] show that the decision to adopt PV is significantly influenced by its previous adoption at neighbourhood level in Switzerland. Graziano et al. [24] revealed similar effects for the United States. Palm [25] found that peers such as relatives, friends and neighbours have a major influence on the decision to invest in PV by confirming that PV systems work as intended and without hassle. The importance of regional and local networks between neighbours and friends for decision-making has also been shown in the acceptance of demand-side management interventions [26]. However, in the context of the federal governance system in Switzerland, questions arise on the role of network effects at subnational level and the possibility of using them to incentivise people at local (district) or regional (canton) level to take up renewable energy technologies [27,28].

Additionally, although proximity effects have been widely discussed in the field of PV, most studies have focused on detecting spatial clusters of PV installations [24,[29], [30], [31], [32]] and, by controlling for the most common variables, homophily, correlated unobservable and simultaneity [29], estimating the extent of the peer effect. However, some authors have claimed that there has been limited analysis on the underlying mechanisms explaining proximity effects [25]. Some other work has focused on understanding the role of proximity effects on the decision-making through surveying the population [25,[33], [34], [35]], but these results are not always contrasted with the spatial distribution of the respondents and PV panels. A set of studies has explored the information circulating between the adopters’ social networks [36] but those networks are rarely integrated in the geographic context [37,38]. Thus, there is a gap in the study of proximity effects that integrated all these angles and offers a comprehensive perspective including perceived influence in the decision and socio-spatial information networks.

Besides proximity effects, other factors have been identified as affecting the decision to adopt an energy technology. Bernards et al. [39] find that income level, average age and household composition are also important factors for energy transition technologies while peer effects seems to affect in the case of PV. Other studies focusing on PV have found proximity effects to be significant for adoption in conjunction with other factors. Bollinger and Gillingham [31] find that higher adoption rates of PV are associated with gender, race, commute length and home repairs. Graziano & Gillinham [29] find that built environment variables, particularly housing density and share of renter-occupied dwellings are significant, as well as the electricity price and the existence of solar programs. Mundaca and Samaita [40] also find significance in subsidies and environmental awareness. Rai et al. [33] highlights the importance of expected financial returns. Considering these findings and the particularities of the context of this region in Switzerland, besides proximity effects, we believe that gender, income level, home ownership and the perception of environmental matters could have an additional impact on the decision.

In the present study, we aimed to better understand the role of proximity factors in the adoption of photovoltaic panels at district level in Switzerland. We focused on the role of geographical and social proximity in the decision to invest in PV. To provide a novel comprehensive perspective to the subject, we approached the study from three angles: (i) the study of the perceived role of proximity effects in adoption through a quantitative survey, (ii) the analysis of the information exchanges regarding PV of those adopting the technology through an ego-network approach (iii) the analysis of the spatial distribution of this network in the geographic space. The following two research questions structured the analysis:

1. How do spatial and social proximity factors influence the adoption of PV?
2. How do these proximity factors relate to other factors that might influence the adoption of photovoltaic panels, such as marital status, income, home ownership and the perception of environmental issues?

This led to the following hypotheses:

1. People tend to adopt PV if their neighbour (spatial proximity) or someone they know (social proximity) has installed one recently.
2. People exchange information on PV panels with others who are spatially close (in terms of distance or sharing a common area) and socially close (friends or people who share common characteristics).
3. Proximity effects are relevant in the adoption of the technology. Other factors, such as gender, income level, home ownership and the perception of environmental matters have an additional significant positive influence on the decision to invest in PV.

The results aim to inform regional and national governments on how to more effectively involve citizens in the energy transition and how cantonal, regional and communal authorities’ power for action could be strengthened in terms of communication and awareness of renewable energies, particularly photovoltaics. In addition, this study offers a new perspective on the analysis of proximity effects by comprehensively analysing it through three angles and looking at the relationships between those.

## 2 Methodology

**Fig. 1:**
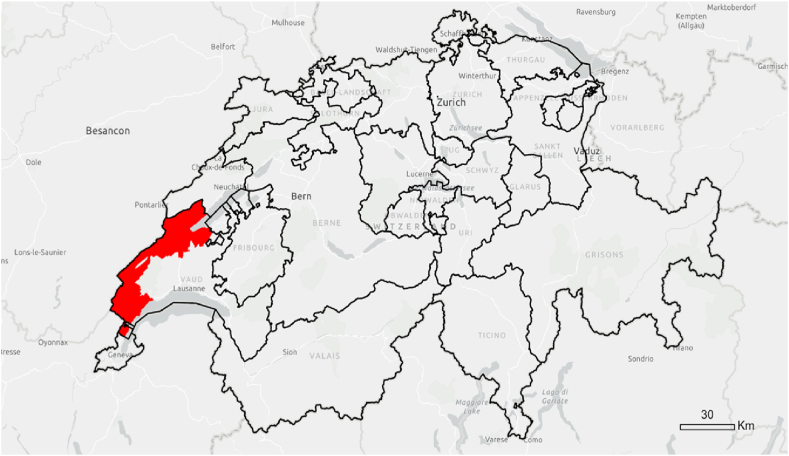
Case study region (red) and Swiss Cantons (black outline). (For interpretation of the references to colour in this figure legend, the reader is referred to the Web version of this article.)

To test these hypotheses, we conducted a survey in two districts of the canton of Vaud, Nyon and Jura-Nord Vaudois (see Fig. 1). These two districts were chosen because of the mix of rural and urban areas, the age structure of the population and voting behaviour in past energy-related ballot polls [41]. Based on the results of the survey, we performed a set of tests to address our hypotheses in a comprehensive way. First, we conducted a logistic regression to identify which factors were the most significant for the adoption of PV. Second, we performed a geographic network analysis to look at the geographic distribution of information networks in the field of photovoltaics. Finally, we used geographic information systems and spatial statistics to understand in more detail the geographic distribution of actors exchanging information on PV. In this section, we present a detailed description of the methods used.

### 2.1 Survey structure

The empirical data set that was used as the basis for our study was collected during fall 2020 through a structured survey sent in the post to a random portion of the population of two districts within the Canton of Vaud. The first part of the survey consisted of socio-demographic questions, including general descriptions of the respondent's situation and their household (age, gender, type and size of household, owned or rented property, etc.). The central part was devoted to questions on the adoption of photovoltaic panels and electric vehicles (not reported in this paper). We asked about motivations for purchasing or implementing the technology, the main source of information, and the influence of people (friends, neighbours, family and colleagues) in deciding whether or not to invest in the technology. In the final part, we asked questions on the relationship between the population and the environment, individual roles in safeguarding the environment, and the economic situation of the household. In total, 1,125 individuals responded to the questionnaire (584 resided in the district of Nyon and 541 in Jura-Nord Vaudois), which represents a response rate of 18.75%. The survey was reviewed and approved by the Swiss Federal Institute of Technology Lausanne (EPFL) human research ethics committee prior to release.

### 2.2 Survey sample

**Fig. 2:**
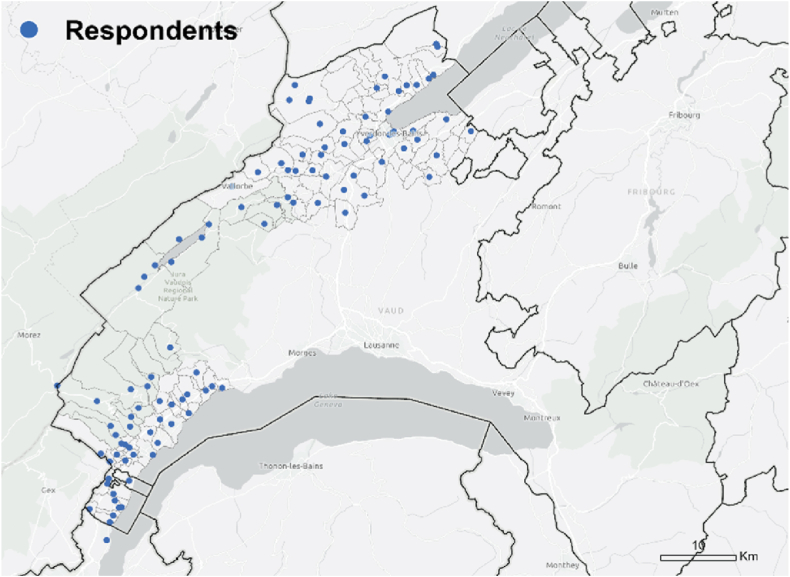
1,105/1,125 respondents located within the case study region.

**Fig. 3:**
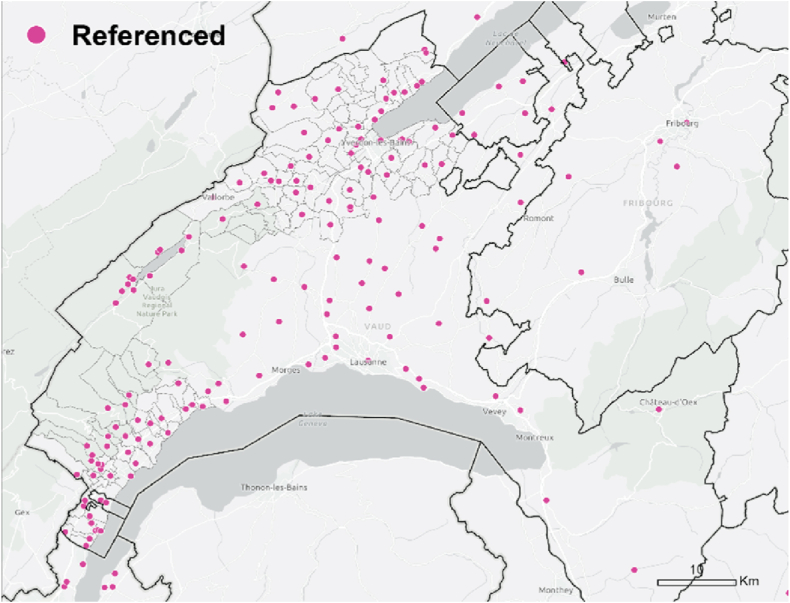
509/696 referenced people located within the case study region.

The sample consists of 1,225 respondents (see Table 1, Fig. 2), 198 of these respondents had adopted the technology (adopters), 443 exchanged information about the technology (information exchangers) and 696 people were identified by the information exchangers when asked if they knew other people who had installed PV panels or if they had advised other people with respect to the installation of PV panels (referenced people, Fig. 3). This last group does not necessarily belong to the sample but are individuals identified by the information exchangers.

**Table 1:** Sample groups.

Sample groups	*n*	%
Respondents	1125	100
Adopters	198	17.6
Information exchangers	443	39.4
Referenced people	696	outside of original sample

Our sample had a majority of male respondents, a higher percentage of people living in a single-family house, more homeowners and wealthier individuals than the cantonal average (Table 2).

**Table 2:** Sample characteristics. Swiss data source: Federal Statistical Office (FSO), Statistical Office Canton of Vaud [41,42].

Socio-demographic characteristics		Sample (%)	Canton Vaud (%)	Switzerland
Gender	Female	33.2	51.7 (2021)	50.4 (2021)
	Male	66.5	48.2 (2021)	49.6 (2021)
Housing type	Single-family houses	50.1	18 (2022)	21 (2022)
	Semi-detached houses	23.5	no data	no data
	Multi-family housing	25.7	56 (2022)	57 (2022)
House ownership	Owners	63.85	29.9 (2019)	42 (2021)
Income	less than CHF 60,000 per year	14.8	77,880 (median 2020)	79,980 (median 2020)
	CHF 60,001 to CHF 88,000	20.4	“	“
	CHF 88,001 to CHF 120,000	24.1	“	“
	CHF 120,001 to CHF 164,999	12.9	“	“
	more than CHF 165,000	11.6	“	“

### 2.3 Logistic regression

Based on the results of the survey, we performed a logistic regression analysis to test the influence of a set of variables (Table 3) on the adoption of PV. Logistic regression is one of the most commonly used multivariate analysis models in statistics. It measures the association between the occurrence of a binary event (explained variable of the type yes/no) and the dependence of the factors that are likely to influence this event (explanatory variables). In contrast to a linear regression based on ordinary squares, logistic regressions can also be run if the assumptions of homoscedasticity, linearity and normality are not met with respect to a dichotomous independent variable (Menard 2002). The regression model (1) follows a general form where β represents the unknown vector of regression coefficients β = (β 0, β 1 , …, β k)^⊤^ and the product x_i_^T^β = β_0_ + x_i1_ β_1_ + x_i2_ β_2_+ … + x_ik_ β_k_ is the linear predictor.

(1) $$P\left(Y=1|X=x_{i}\right)=P\left(Y_{i}=1\right)=\frac{\exp\left(x_{i}^{t}\beta \right)}{1+\exp\left(x_{i}^{t}\beta \right)}=\frac{1}{1+\exp\left(-x_{i}^{t}\beta \right)}$$

**Table 3:** List of variables integrated in the regression model.

Variable	Variable type	Description
Age	Continuous	1 to 100
Sex	Discrete	male or female
Household composition 0-17	Categorical	number of people
Household composition 18-35	Categorical	number of people
Household composition 36-50	Categorical	number of people
Household composition 51-65	Categorical	number of people
Household composition 66+	Categorical	number of people
Home ownership	Discrete	yes or no
Type of dwelling	Categorical	individual house, terraced house, apartment
Length of ownership	Categorical	categories of years (5 years)
Relationship with the neighbours	Categorical	5-scale Likert
Identification with the neighbourhood	Categorical	5-scale Likert
Responsibility for energy expenditure	Categorical	4-scale Likert
Interest in the environment	Categorical	4-scale Likert
Exchanges about environmental issues	Discrete	6-scale Likert
Panel installation by people you know	Discrete	yes or no
Installation of PV panels by neighbours	Discrete	yes or no
Exchange with neighbours	Categorical	6-scale Likert
Advice given in the past	Discrete	yes or no
Consideration of own impact on the environment	Categorical	6-scale Likert
Impact of habitudes on the environment	Categorical	6-scale Likert
Concern with climate impact of energy use	Categorical	6-scale Likert
Support for environmental protection	Categorical	6-scale Likert
Priority given to environmental issues	Categorical	6-scale Likert
Professional status	Categorical	fully employed, partially employed, house man/wife, in education, without work, retired
Civil status	Categorical	single, married, divorced or widowed
Income level	Categorical	5 categories from < CHF 60,000 to CHF 165,000 >
Educational level	Categorical	10 categories from no formal education to university degree

Before performing the logistic regression, we controlled for collinearity by calculating the correlation of the variables included with the Pearson's product-moment correlation coefficient, obtaining coefficients <0.7, and we measured the Variance Inflation Factor (VIF), obtaining values < 5 (Appendix A2). To conduct this test and the logistic regression, we used the IBM SPSS statistics package and the statsmodels python package.

### 2.4 Geospatial analysis of information exchange networks

Based on the demonstrated importance of personal contacts and proximity for the decision to invest in photovoltaics, our next step was to conduct an analysis that focused on the sharing of information about the installation of photovoltaic panels in our sample. Specifically, we looked at the geographical distribution of these information networks, the role that individual actors play with regard to the dispersion of information and whether or not geographical proximity plays a significant role in the sharing of information.

We decided to approach the analysis of proximity effects at different scales, starting from European level and ending by looking at the short straight distances between respondents (in kilometres) within the case-study regions. For this analysis, we focused on survey respondents who had an exchange with another actor about PV panels (*information exchangers*) and those who were referenced by them (*referenced people*; see Table 1). In this group, we include adopters of the technology and non-adopters. We obtained the geographical location of the respondents using the MMQGIS geocoding plugin [43] based on the addresses provided in the responses of the survey and the OSM/nominatim base. We successfully geolocated 1,105/1,125 respondents and 509/696 *referenced people*.

#### 2.4.1 European and national scale

To look at the geographical distribution of information networks in the field of photovoltaics at European and national scale, we performed a geographical network analysis using Gephi and Tulip software. The network analysis allowed us to look at the structures of information exchange between respondents and to see whether there was geographical dependency according to the residence of the respondents. We looked at basic measures of density, centrality and modularity. Density is calculated as the ratio between the existing number of ties and the maximum possible number of ties in a graph, and indicates how many of the potentially existing connections are actually activated in the network [44]. Centrality depicts the importance of a single node in a network in terms of number of connections, and indicates the overall concentration of connections within the network [45]. Finally, modularity measures the tendency to form modular subgroups or communities in a graph, where a group of nodes has denser ties within than to nodes outside the group (see Ref. [44] for more details). Higher modularity is associated with a higher tendency of various approaches to a problem to be developed within the network and, following, a higher chance of a contest of ideas to emerge [46]. Additionally, we analysed the embedding of the network in the geographic contexts and observed patterns in the geographic distribution of the links. To visualize the network, we used the Yifan Hu algorithm, which combines a force-directed model with a graph coarsening technique (multilevel algorithm) to reduce the complexity of the representation [45].

#### 2.4.2 Cantonal and regional scale

To bring the analysis to a smaller geographic scale, we studied the relationships between the respondent's location and the geographic context using ArcGIS Pro, geographic information system (GIS) software from Esri. We were particularly interested to see whether geographic clusters of information exchange exist at cantonal scale and whether sub-regions could be identified in the two districts where this exchange is still struggling to appear. To conduct these types of analysis, we used density cartographies and spatial statistics to detect clusters of respondents and compare them with the current location of existing PV installations, the degree of urbanisation of the region, population density, housing density, access to services.

Specifically, we started analysing the spatial concentration and distribution patterns of *respondents*, *referenced people* and PV installations in the region using a global statistics measure: the average nearest neighbour. This tool averages all the distances between each feature's centroid and its nearest neighbour. The features' distribution is dispersed or concentrated depending on their relationship with a hypothetical random distribution value [47,48]. Then, we conducted a spatial hotspot analysis, which statistically identifies areas of concentration of high values (hotspots) and areas of concentration of low values (cold spots) among a set of weighted features using the Getis-Ord Gi* statistic (2, 3, 4) [49,50], considering the location of the *respondents* of the survey and the *referenced people*.

(2) $$G_{i}^{*}=\frac{\sum_{j=1}^{n}\omega_{i,j}x_{j}-\bar{Χ}\;\sum_{j=1}^{n}\omega_{i,j}}{S\sqrt{\frac{\left\lfloor n\sum_{j=1}^{n}\omega_{i.j}^{2}-{\left(\sum_{j=1}^{n}\omega_{i,j}\right)}^{2}\right\rfloor }{n-1}}}$$

Where $x_{j}$ is the attribute value for feature j , $\omega_{i,j}$ is the spatial weight between feature i and j, n is equal to the total number of features and:

(3) $$\bar{X}=\frac{\sum_{j=1}^{n}x_{j}}{n}$$

(4) $$S=\sqrt{\frac{\sum_{j=1}^{n}x_{j}^{2}}{n}-{\left(\bar{X}\right)}^{2}}$$

The output of the Gi* statistic is a z score for each future. A concentration of high values (hotspot) will show highly statistically significant z-scores while a concentration of low values (cold spot) will show statistically significant negative z-scores.

Finally, the proximity of respondents to existing PV was measured using an alternative approach, by calculating the number of PV installations within a given distance from the *respondents* of the survey and the *referenced people*. The measure was run using two distance thresholds selected based on PV diffusion literature in European regions: 500 m, 1 km [30,51] and 4 km [52]. Due to non-parametric distributions, a Kruskal-Wallis test was used to test the significance of the differences between information exchangers and referenced.

#### 2.4.3 Municipal and short-distance scale

After analysing the characteristics of the network and the geographic distribution of the respondents in relation to existing PV installations, we took a step further in connecting the network of actors to the geographic context. To achieve this, we used an Exponential Random Graph Model (ERGM) (5) to study the probability that a presence of a tie in the network is affected by (i) straight line distance, (ii) belonging to the same municipality and (iii) belonging to the same postcode. ERGMs are a family of statistical models used in social network analysis that enable network pattern recognition [53]. Their goal is to “describe parsimoniously the local selection forces that shape the global structure of a network” [54]. As in the case of logistic regression, they predict the probability that two nodes are connected by a tie. However, in this case, they do so by comparing it with an Exponential Random Graph, considered as the null hypothesis. For this study, we used the ERGM network of the statnet R package powered by the Markov Chain Monte Carlo (MCMC) algorithm.

(5) $$P\left(Y=y\right)=\frac{\exp\left(\Theta^{\prime }g\left(y\right)\right)}{k\left(\Theta \right)}$$

Y is the random variable for the state of the network (with realisation y), g(y) is a vector of model statistics for network y, θ is the vector of coefficients for those statistics and k(θ) represents the quantity in the numerator summed over all possible networks [55]. Although the ERGM package includes 150 terms, we only used the following terms. (i) Edges is a parameter that reflects the odd of every link of the network of existing, also called the density of the network. It was used as a baseline value for our analysis. (ii) Nodematch, with the attribute diff, counts how many extremities of links have matched attributes. (iii) Edgecov (edge covariate) refers to the attributes of the edges of the network, in this case, all the distances between actors in the network. These variables were assessed alone and in conjunction to test their effect on the creation of a link. The ERGM only considers the probability of having a link within the existing nodes, which are already spatially concentrated in the case study regions, as we have seen before. Therefore, within the spatial clustering that we have already detected, we tested at a finer grain whether there are also correlations between living at shorter distances to each other. To contextualise these results, the local characteristics of areas with a high concentration were compared with those in the case study region.

## 3 Results

### 3.1 Descriptive statistics

A total of 198 out of 1,125 respondents in the sample have installed a photovoltaic installation on their house, which represents 17.6% of respondents. The average power of the installation was 7 kWp. This corresponds to approximately 45 m^2^ of solar panels [56]. Twenty-five people (2.2%) had supplemented their installation with battery storage, and 13 were part of a self-consumption community (1.2%), which is above the Swiss mean. Across all groups, more than half of the respondents (413 out of a total of 823 respondents who answered this question) had considered investing in PV in the past.

### 3.2 Ownership, neighbours and social connections are relevant factors for adoption

Four variables were found to be significant in the adoption of PV panels (see Table 4). These were home ownership, panel installation in the social group, panel installation by neighbours and professional status. Home ownership and panels in the social group are the most significant variables (p < 0.01). Being a homeowner increases the probability of installing PV panels by 271%, while panel installation in their social group leads to an 89% increase in probability of installation. Having neighbours who have installed PV panels leads to a minor probability increase of 0.05%. Finally, the current level of professional engagement also has a significant impact on the probability of installing PV panels. The counterintuitive odds-ratio of <1 is due to the formation of the categories, starting with the lowest and going towards the highest (i.e. university) education (see Table 4). Other variables, such as environmental attitude, had no significant effect on the probability of installing PV panels. The omnibus test of model coefficient showed a p < 0.001, determining a statistically significant model.

**Table 4:** Results of the logit regression on the probability of setting up a photovoltaic installation. For complete analysis review the supplementary material.

Explanatory variables	B	S.E.	Wald	Sig.	Exp (B)
Age	,008	,006	1,994	,158	1,008
Sex	,030	,069	,188	,665	1,031
Household composition 0-17	-,071	,077	,855	,355	,931
Household composition 18-35	,032	,057	,320	,571	1,033
Household composition 36-50	-,006	,023	,078	,779	,994
Household composition 51-65	,004	,011	,137	,712	1,004
Household composition 66	-,006	,006	1,017	,313	,994
**Home Ownership (A04)**	**1,312**	**,196**	**44,757**	**<,001**	3,712***
Type of dwelling	-,005	,007	,505	,477	,995
Ownership duration	,016	,028	,319	,572	1,016
Relationship with the neighbours	,001	,008	,030	,861	1,001
Identification with the neighbourhood	,005	,003	2,334	,127	1,005
Responsibility of energy expenditure	-,002	,082	,000	,984	,998
Interest in the environment	,006	,006	,786	,375	1,006
Exchanges about environmental questions	,004	,015	,076	,783	1,004
**Panel installation by people you know**	**,637**	**,139**	**21,001**	**<,001**	1,891***
**Installation of PV panels by neighbours**	**,005**	**,002**	**5,420**	**,020**	1,005*
Exchange with neighbours	,003	,005	,318	,573	1,003
Advice given in the past	-,204	,168	1,471	,225	,815
Consideration of own impact on environment	-,004	,006	,462	,497	,996
Impact of habits on environment	,004	,007	,296	,586	1,004
Preoccupation with climate impact of energy use	,015	,009	3,183	,074	1,015
Support of environmental protection	-,011	,009	1,418	,234	,989
Priority given to environmental issues	,003	,006	,294	,588	1,003
**Professional status**	**-,010**	**,004**	**5,173**	**,023**	,990*
Civil status	,007	,008	,673	,412	1,007
Level of revenue	,001	,002	,091	,763	1,001
Level of education	,000	,007	,001	,977	1,000
Constant	−2,530	,594	18,138	<,001	,080

*** = significant at the 1% level * = significant at the 5% level.

### 3.3 Network structure

In the network, we had an overall number of 1,821 nodes, the 1,125 survey *respondents* plus 696 *referenced people*, which resulted in 696 connections in the graph. Of the respondents to the survey, 682 did not report any connection with other people regarding the exchange of information on photovoltaic panel installations. For the rest of the *respondents*, the number of connections rose from 1 to 18, with a very small number of people taking over the role of knowledge diffusers in the network. A total of 47 individuals in the network shared 3 or more connections with others.

**Fig. 4:**
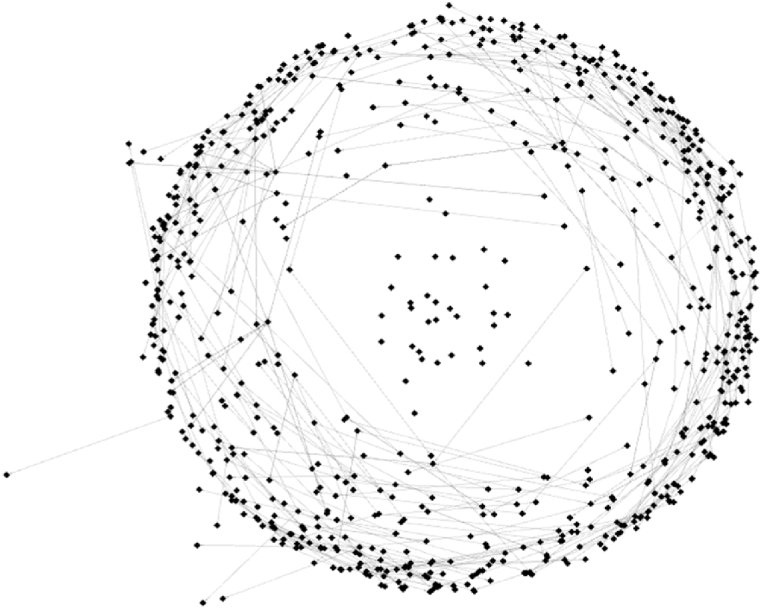
The overall knowledge exchange network of actors in the two districts (own graphic generated with Gephi).

Overall, the network was very sparse, which reflects the scattered flow of information and the individualised points of reference indicated by the respondents. The low level of overall connectivity is of course also a result of the chosen method, since we might have missed out on some of the strongly connected people due to the random choice of respondents. It had an overall degree of centrality of 0.382 (on a scale from 0 to 1), which means that the network overall was not very centralised and the connections were quite evenly spread (see Ref. [44] for more information on the interpretation of network metrics in social science). This is in line with the fact that most information exchangers indicated one to three connections with whom they have exchanged information. The average path length was 1.305 (which means that on average every node within the network could be reached in 1.305 steps from any other node in the network), and there were hardly any subgroups in the network, with a modularity coefficient of 0.996 (a modularity coefficient of 1 equals a network with a totally equal distribution of links across the network). Fig. 4 shows the network of information exchanges based on the Yifan Hu projection [45].

### 3.4 Linguistic clustering at national scale

**Fig. 5:**
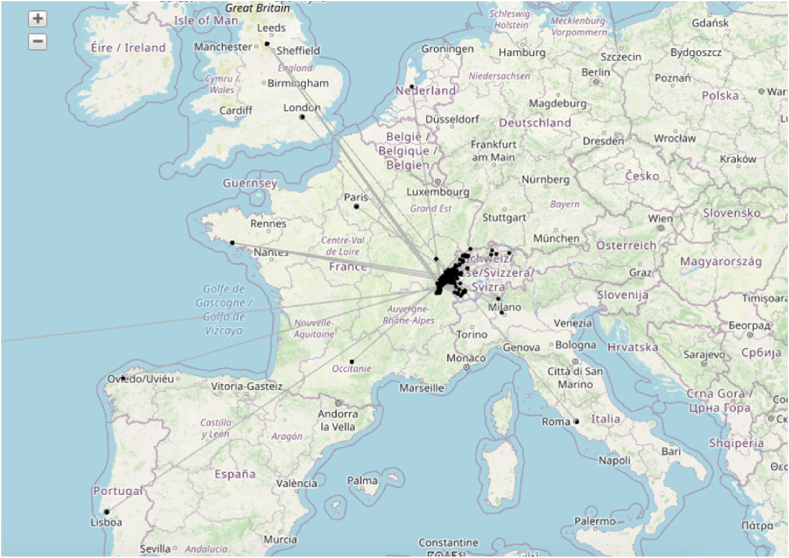
Concentration of the information exchange in the western part of Europe (own graphic generated in Tulip software on the basis of Open Street Map).

When we examined the spatial distribution of information exchanges on PV at supranational scale, we detected that the survey respondents were in direct contact with peers in many western European countries (mainly France, but also the UK, Italy, Spain, Portugal and the Netherlands). Very few exchanges took place beyond these restricted geographical boundaries, with one single connection to North America and no connections to the rest of the world (Fig. 5).

**Fig. 6:**
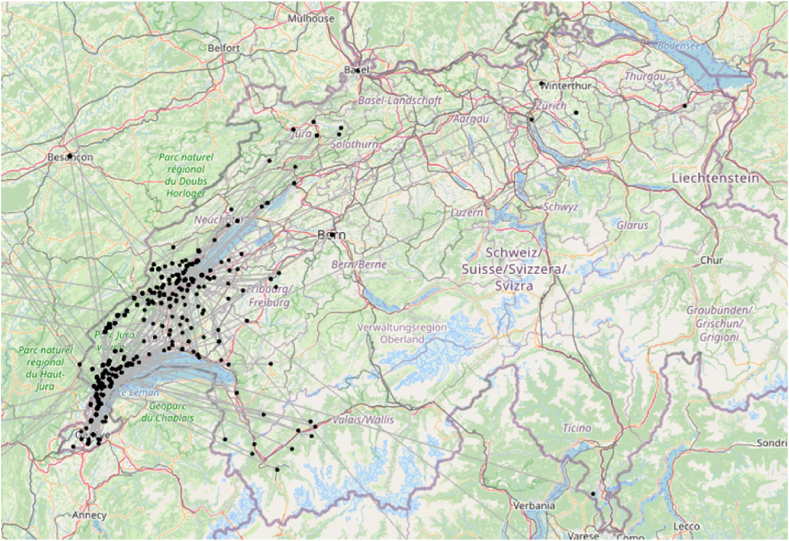
The clustering of the information exchange network in the western part of Switzerland (own graphic generated in Tulip software on the basis of Open Street Map).

When we examined the national scale, we detected strong geographical clustering around the two case study areas of Nyon and Jura-Nord vaudois (Fig. 6). A total of 73% of the *referenced people*, people referred to in the survey as connections who have installed a photovoltaic panel, were located within the case study areas.

We also found a clear effect of the linguistic barrier in Switzerland, with most connections outside the canton of Vaud focused on the canton of Geneva and the other cantons of the French-speaking part of the country, as well as France. An examination of the distribution of links within the network showed a predominance of links purely within French-speaking municipalities and regions of Switzerland, with only a small share of connections crossing the linguistic barrier in Switzerland (1.4% of *referenced people*) or linking actors internationally (3.3% of *referenced people*).

In the relation between the overall distribution of the *referenced people*, named by the survey respondents as their point of contact to exchange information on PV installations, no clear relation can be established between the degree of PV installations at the level of individual municipalities in the canton of Vaud and the number of people referred to in those municipalities. The same is true for the potential of those municipalities with respect to PV power generation.

### 3.5 Clustering of information exchangers at cantonal and regional scale

We found different patterns at cantonal scale. All *Respondents* are located within the study areas due to the sample selection, while 27% of *referenced* expand beyond that boundary, mostly remaining within the Canton of Vaud (Fig. 2). The spatial statistical analysis confirmed the patterns expected in an urbanised environment. The z-score (−73.06) of the nearest neighbour algorithm indicates that the *information exchangers* and *referenced people* were not randomly distributed in the territory but clustered. The observed mean distance (47 m) between the information exchangers was lower than the expected one (3,590 m), which suggests that there were geographically concentrated clusters. In the case of the *referenced people*, although the observed mean distance was lower, the difference was much smaller (1,143 m to 67,193 m), which suggest that the clusters were less concentrated than in the case of *information exchangers*, a pattern that aligns with the sample selection process.

**Fig. 7:**
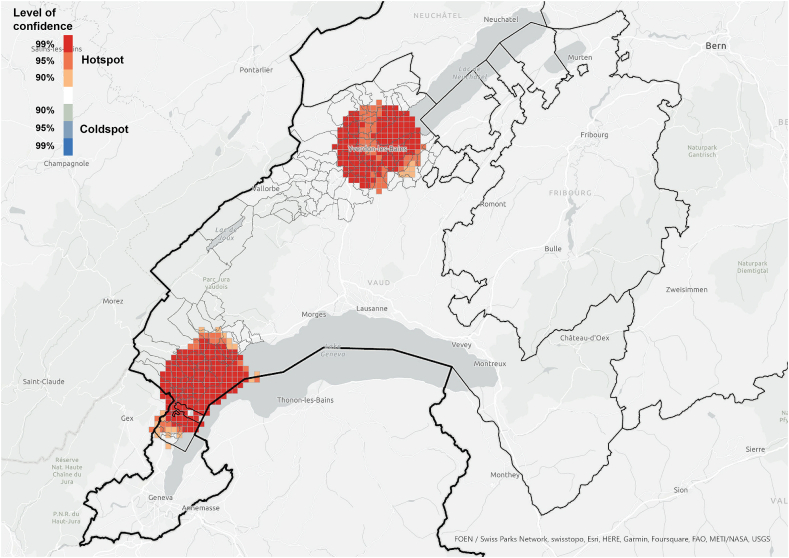
Hotspot analysis in a fishnet grid of 1-km cells at cantonal level of *information exchangers*.

**Fig. 8:**
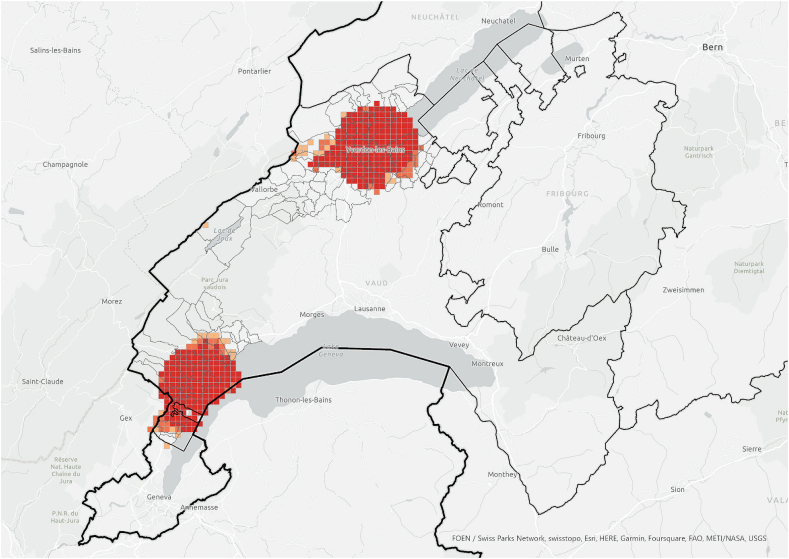
Hotspot analysis in a fishnet grid of 1-km cells at cantonal level of *referenced people*.

**Fig. 9:**
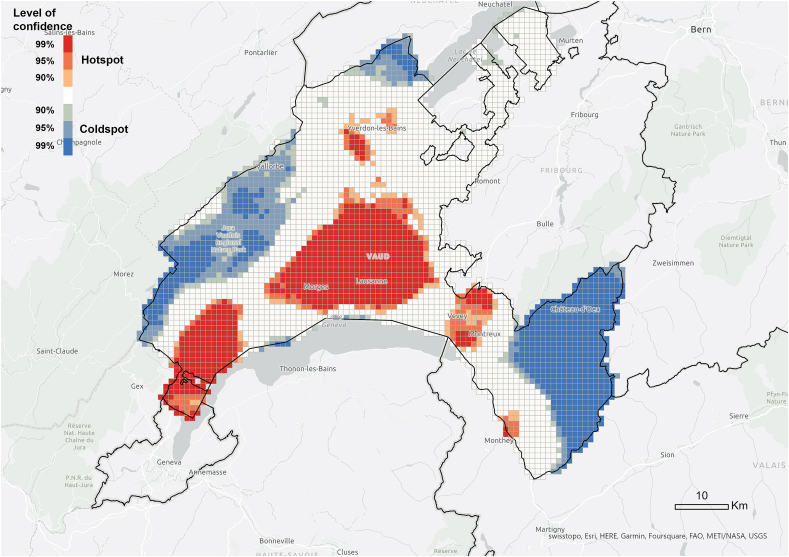
Hotspot analysis in a fishnet grid of 1-km cells at cantonal level of the location of PV installations.

**Fig. 10:**
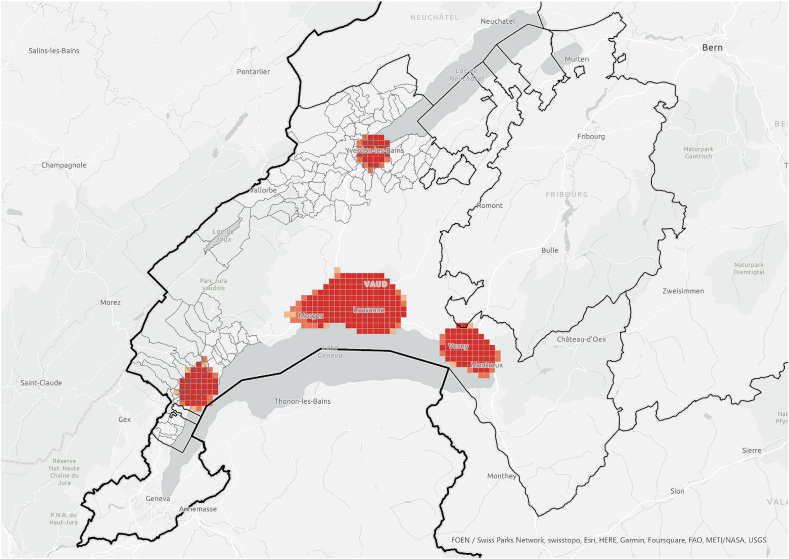
Hotspot analysis in a fishnet grid of 1-km cells at cantonal level of population density.

The hotspot analysis at cantonal level showed that, most of the information networks were concentrated within the two districts in which the survey was conducted, which supports the argument of the importance of spatial proximity. Although *referenced people* were not only located within the boundaries of the study area and expanded throughout the canton (Fig. 3), they were spatially concentrated in the same areas where we find the information *exchangers* (Fig. 7, Fig. 8). These areas did not align with the hotspots of PV installations at cantonal level (Fig. 9), which rather align with population distribution (Fig. 10). Particularly, the region around the city of Lausanne was characterised by a high spatial concentration of PV adoption but did not show any significance for both *information exchangers* and *referenced people.* Thus, referenced people are rather concentrated around the people that exchanged with them instead of being located in areas with high concentration of PV. When only considering the referenced outside the case study area, they are concentrated in Lausanne, the largest city in the Canton.

**Fig. 11:**
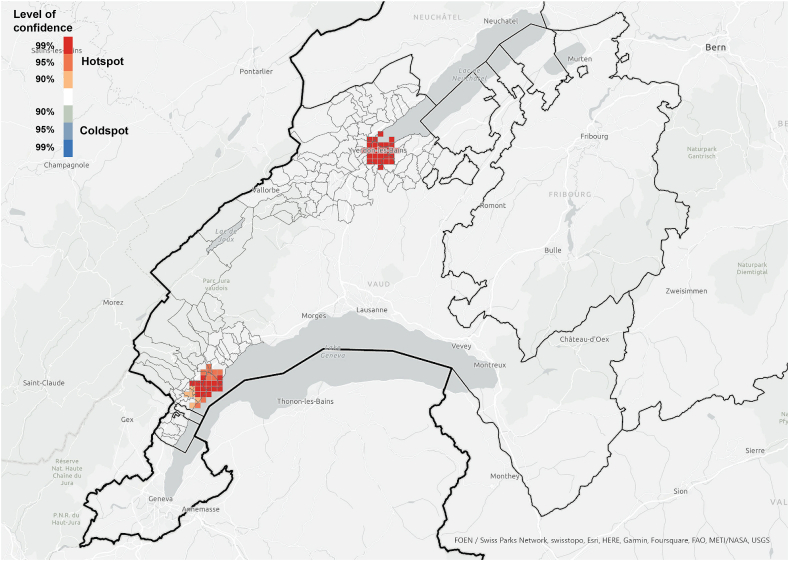
Hotspot analysis in a fishnet grid of 1-km cells at the case study level of *information exchangers*.

**Fig. 12:**
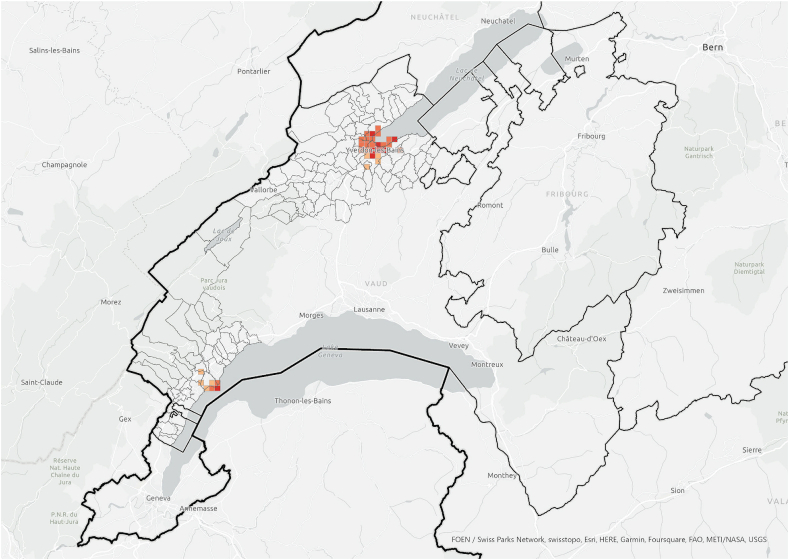
Hotspot analysis in a fishnet grid of 1-km cells at the case study level of *referenced people*.

**Fig. 13:**
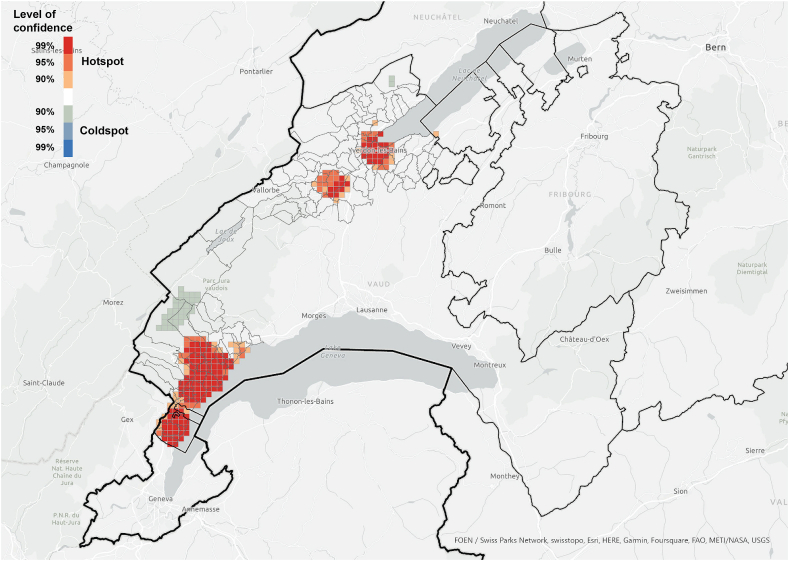
Hotspot analysis in a fishnet grid of 1-km cells at the case study level of the location of PV installations.

When the hotspot analysis was conducted at case study regional level, *information exchangers*, *referenced people* and PV installations showed aligned hotspots (Fig. 11, Fig. 12, Fig. 13), located in the urban centres of Nyon and Yverdon-les-Bains for the three groups and, additionally, in Orbe for PV installations. These observations indicate that both *information exchangers* of the survey and the actors *referenced* by them were concentrated in areas with high PV presence. We can also see that *information exchangers* and *referenced people* hotspots were within the PV hotspots but more concentrated in the most populated areas (appendix A5-1).

**Fig. 14:**
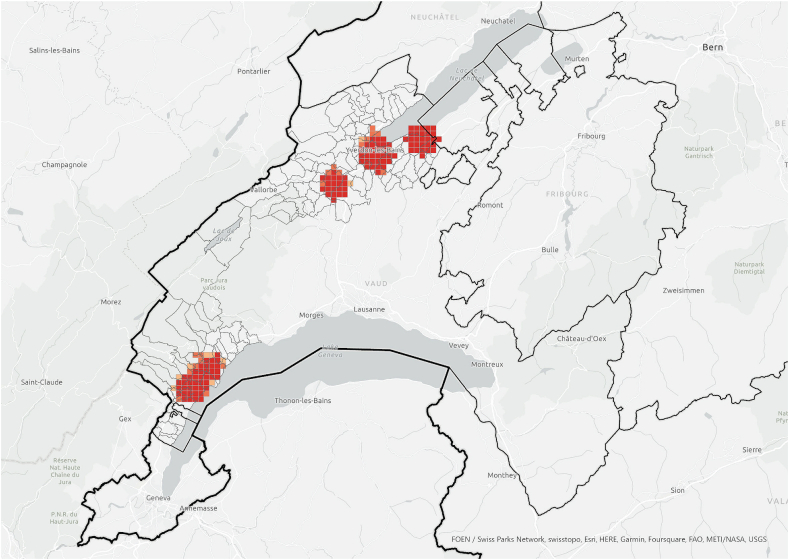
Hotspot analysis in a fishnet grid of 1-km cells at the case study level of the total power of PV installations.

**Fig. 15:**
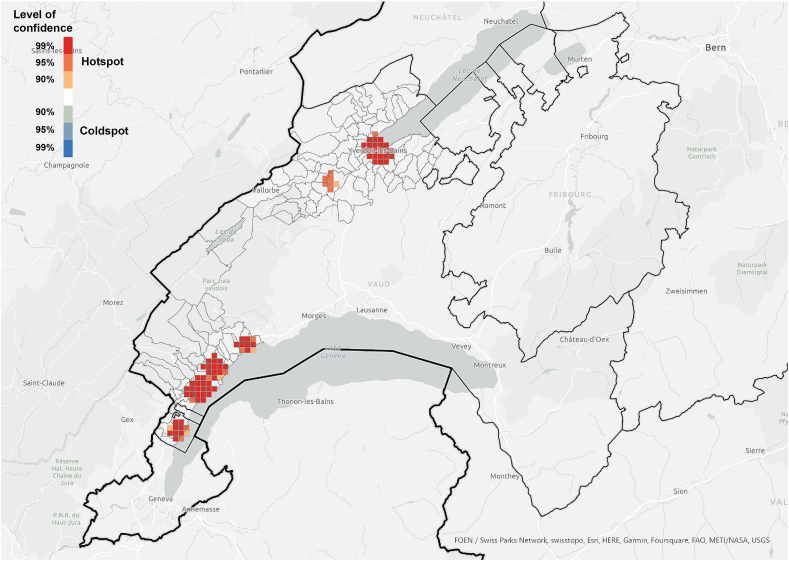
Hotspot analysis in a fishnet grid of 1-km cells at the case study level of population.

**Fig. 16:**
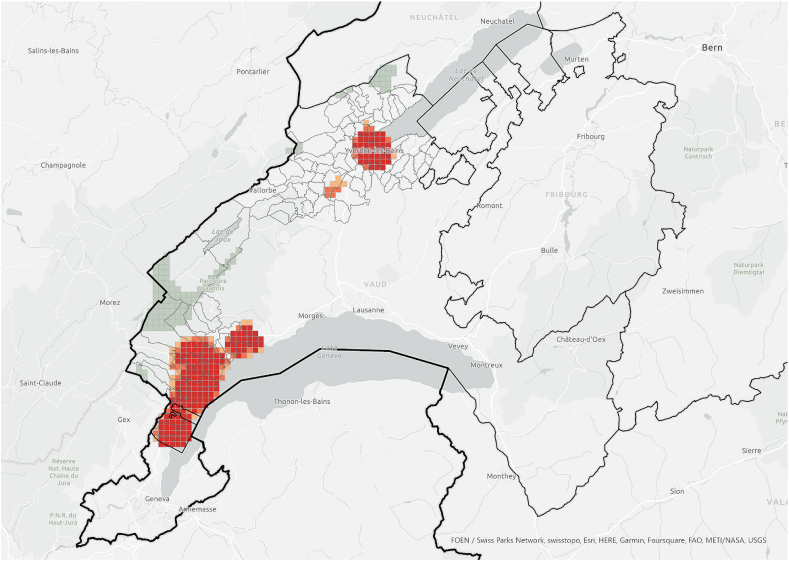
Hotspot analysis in a fishnet grid of 1-km cells at the case study level of housing units.

In general, these areas are the ones with highest population density, housing density and better access to neighbourhood services, which is visible both in the hotspot maps (Fig. 11, Fig. 12, Fig. 13, Fig. 14, Fig. 15, Fig. 16 and appendix A5-9) and the kernel density maps (Appendix A5 6–8). Once looking in detail, we can see additional patterns. On the one hand, PV installations (Fig. 13, Fig. 14) align better with housing units (Fig. 16) than population density (Fig. 10 left), as they both have larger hotspots in the territory. However, the PV hotspot in Orbe has higher values and is more extended in the territory than the housing one. On the other hand, *information exchangers* (Fig. 11 left) are concentrated in the most populated areas (Fig. 15 left) rather than the areas with more housing units (Fig. 16 right), with a tendency to focus on the two most important urban centres of the region: Nyon and Yverdon-les-Bains.

**Fig. 17:**
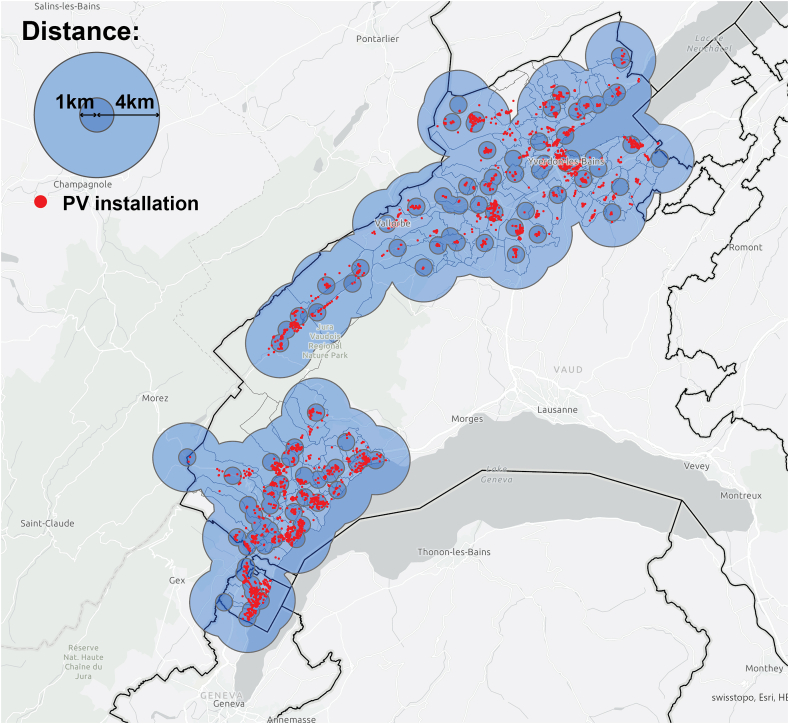
A total of 2,272/3,210 (71%) of PV installations within 1 km of the *information exchangers* 3,205/3,210 (99.8%) and PV installations within 4 km of the *information exchangers*.

**Fig. 18:**
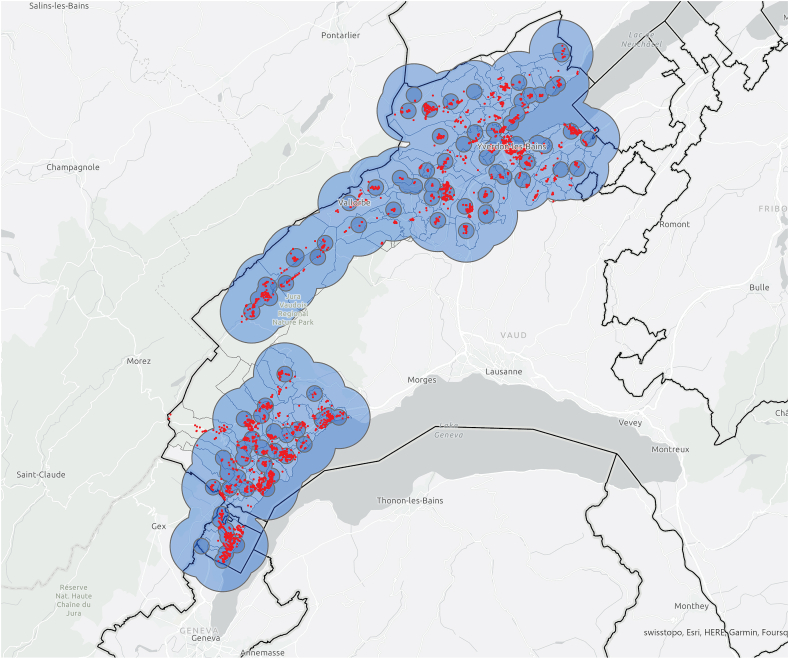
A total of 2,216/3,210 (69%) of PV installations within 1 km of the *referenced people* 3,197/3,210 (99.6%) and PV installations within 4 km of the *referenced people*.

When the number of PV installations at 1 km and 4 km from the *information exchangers* was calculated (Fig. 17, Fig. 18), the results also supported the previous findings, with a majority of PV installations located close to the *information exchangers* (40% within 500 m, 71% within 1 km and 99.8% within 4 km) and to the *referenced* actors (36% within 500 m, 69% within 1 km and 99.6% within 4 km). *Information exchangers* have a mean of 18 PV installations within 500 m of their home (σ = 15.6) 53 within 1 km (σ = 32.6) and 270 within 4 km (σ = 103.4). *Referenced* have a mean of 18 PV installations within 500 m of their home (σ = 15.6) 53 within 1 km (σ = 32.6) and 235 within 4 km (σ = 111.3) (Table 5). The number of installations around referenced people seem to be significantly higher than for information exchangers. This would be aligned with a situation in which the referenced may have firstly been influenced by their environment, adopted PV themselves and, then, through active peer effects, share this experience with the information exchangers.

**Table 5:** Mean number of PV panels around *information exchangers* and *referenced*.

	Information exchangers	Referenced within case study region	All referenced	Information exchangers vs. all referenced Kruskal-Wallis test
500 m	18 (σ = 15.6)	16 (σ = 13.8)	24 (σ = 20.7)	14.1 (p < 0.01)
1 km	53 (σ = 32.6)	46 (σ = 32)	71 (σ = 47)	60.6 (p < 0.01)
4 km	270 (σ = 103.4)	235 (σ = 111.3)	484 (σ = 277.4)	298.7 (p < 0.01)

### 3.6 Belonging to the same municipality matters more than distance

In terms of straight-line distance in km, the ERGM results (Table 6) did not show any significant relationship between proximity and the probability of having a link. However, the results were significant (p < 0.05) for two codes belonging to three municipalities: Azier-Le-Muids, Rolle and Yverdon-les-Bains, and for the three postcodes of these municipalities. All these were very close to the urban centres detected before as concentrations of PV installations, *information exchangers* and *referenced people*. The results also showed some significance for surrounding municipalities. The three municipalities with significant results had an intermediate level of urbanisation [57], the highest in the case study area. They also had higher population density levels and were important urban centres within the case study region.

**Table 6:** Significant results of the ERGM model.

		Estimate	Std. Error	MCMC%	Z value	PR (>|z|)
Link - Postcode	1180 1273 1400	1.51037 2.04318 0.59030	0.70908 0.71016 0.15291	0 0 0	2.130 2.877 3.860	0.033167 * 0.004014 ** 0.000113 ***
Link - Municipality	Arzier-Le Muids Rolle Yverdon-les-Bains	2.04228 1.50947 0.53882	0.71015 0.70907 0.17864	0 0 0	2.876 2.129 3.016	0.00403 ** 0.03327 * 0.00256 **
Link - Distance		−7.238e-08	1.032e-07	0	−0.701	0.483

Significance levels: *** = 0 ** = 0.001 * = 0.05.

## 4 Discussion and recommendations

### 4.1 Peer effects matter

Regarding our first hypothesis, we can confirm that people tend to adopt PV if they know of others socially or spatially close to them who have already installed PV. Overall, the fact that neighbours have put up PV panels has a statistically significant impact on the likelihood of survey respondents in the two surveyed regions of Switzerland to put up PV panels themselves. Having people in their social group who have put up PV panels also plays a significant role in a person's decision to invest in PV. A total of 40.4% of respondents know at least one person in their personal network who has already installed photovoltaic panels. These findings are in line with many previous studies that looked at the influence of spatially close surrounding installations on the adoption of PV in Switzerland [23,30] in other parts of Europe [58] and the US [29,[31], [32], [33]].

We can also confirm the second hypothesis that people exchange information on PV panels with others who are socially close (friends and family, and those who mostly share the same language) and spatially close (neighbours and people living in the same geographic area). Actors seem to value the information exchanged between peers when they make a decision, which supports other studies [25,31] that emphasize the importance of active peer effects (direct interpersonal contact), particularly in the exponential stage of the S-shaped diffusion curve [29]. If we look at the 19.4% of respondents who have already advised someone else on the installation of photovoltaic panels, most of these people have advised a friend (55%, multiple answers possible), followed by neighbours (42.7%) and family members (32.1%). These results are in line with Palm's [25] findings in Sweden, in which active peer effects mostly take place through existing social networks (friends, colleagues and relatives) and with people who have a close relationship.

Spatial proximity seems to matter at regional level for these exchanges to happen, in terms of belonging to the same geographic area (municipality or postcode) but not in terms of straight distance. Other studies have found spatial peer effects to be concentrated in neighbourhoods [29]. This is in line with the reports of our survey respondents about their neighbourhood sense of belonging. The majority of respondents see themselves as being well integrated in the context of their neighbourhood. A total 53.6% of the respondents speak of a close or very close relationship with their immediate neighbours, which translates into exchanges at least once a week. A total of 31.5% indicate that they have a neutral relationship with monthly exchanges, while 13.6% see themselves as distant or very distant from their neighbours. A total of 44.7% of respondents report a strong or rather strong identification with their neighbourhood, compared to 20.6% who identify little or no identification with their neighbourhood. A total of 28.3% are in a neutral position, 6.5% did not answer the question.

Regarding the third and final hypothesis, home ownership and the current level of professional engagement (i.e. whether or not you work 100%) have a significant positive influence on the decision to invest in PV [29]. also found the proportion of home ownership to be significant in their PV adoption studies. In contrast, gender, income level, the perception of environmental matters, along with a number of other factors such as length of ownership of the house or the fact that the person is responsible for energy expenditure have no statistically significant influence on putting up PV panels.

This study is particularly relevant because it combines an observational analysis on the location of PV installations and an inquiry into the drivers of adoption through a survey. In this way, proximity effects are not only identified through spatial clusters of PV panels, but their relevance is also asserted in the respondents’ answers that identify the influence of neighbours and close contacts. This approach is a first step to address the limitations observed by Palm [25], who claimed that, often, even if clusters of PV installations are detected, the proximity effect mechanisms are rarely explored. It also addresses the concerns of Brugger & Henry [36], who urged people to consider network structures when they promote the diffusion of PV to avoid distributional problems among the population.

### 4.2 The relevance of geographic proximity varies depending on scale

The results of the survey seem to confirm both the potential of active (word of mouth) and passive (visibility) peer effects [32], as respondents report having exchanged information and having seen their neighbours’ installations. When we examine in more detail at the spatial distribution of the information network nodes, we find different patterns at the various scales analysed. Previous studies addressed the scale of studying peer effects and indicated threshold limits [30,51,52], the distance-based diminution [29] or even specific stronger scales, such as street level [31]. The collection of survey data and building an information network allows us to offer complementary insights on how these peer effects can act at different scales.

At European scale, we can see that survey *information exchangers* are more in contact with western European countries than other areas of the world. Within the Swiss national boundaries, we find a linguistic clustering, with *information exchangers* having more exchanges with the French-speaking cantons and municipalities. These results support the findings of Baranzini [30] and Carattini [59], who highlight the relevance of linguistic boundaries in the diffusion of PV in Switzerland.

At cantonal level, we find an expected statistical clustering of respondents and *referenced people* in the regions of study and a mismatch with the location of PV installations, which are rather concentrated in the most important urban areas within the canton. However, when only the case study regions are considered, the three clusters align: *information exchangers*, *referenced people* and PV installations. In the region of Nyon, the importance of inner-regional connections could be partially explained by the high share of PV installations. However, this is not the case for the larger area of the Jura-Nord Vaudois, which has a lower PV concentration.

Finally, belonging to the same municipality and postcode is significant in the generation of an information exchange link, while straight distance between actors is not significant. The municipalities with high significance in the creation of a link are the most urbanised in the case study region and have a high share of PV installations. Therefore, the pattern of spatial proximity in the adoption of PV could be the result of general population density concentration in urban centres, which explains both the higher number of *information exchangers* and *referenced people* and the higher number of PV installations. These results align with the studies of Graziano [29] in the US, which found that PV systems were absorbed faster in urban environments than suburban ones. Regardless of the concentration in urban areas, it is still significant that municipal boundaries seem to have a more important effect than straight-line proximity, pointing at the impact of administrative divisions of the territory on proximity effects and, thus, on the diffusion of PV.

### 4.3 Multidimensionality of geographic proximity

When we look at the specific distribution of nodes in the information exchange network based on the survey results from the two districts analysed, a number of key elements regarding the importance of geographic and cultural proximity in the exchange of specific information in relation to photovoltaic installations could be identified. Besides the pure geographical straight distance, additional aspects such as administrative boundaries, shared language or urbanisation degree seem to play a role in the way the information network presents itself. The results show a higher density of exchanges between people living in French-speaking areas than other surrounding languages, with this variable even superseding national boundaries. Respondents seem to exchange more with people living in France that people living in Switzerland in the German-speaking region. Additionally, the probability of having a link is ties to sharing location in the same municipality, rather than geographic distance. This further supports the assumption that certain types of geographic proximity play a significant role in the exchange of place- and context-specific information regarding the installation of photovoltaic installations. Therefore, future studies of proximity effects should rather focus on identifying the relevant proximity factors of the context rather than only considering straight distance. Policies to support the diffusion of PV should also identify the significant boundaries of the area, be it language based, tied to the presence of an energy utility or the area of influence of a supplier. In this way, specific policies can better target the local needs and rapidly activate existing information networks on the topic.

The geographic analysis also highlights the need to develop spatial proximity measures that address the dangers of self-selection of peers (homophily), correlated unobservables, and simultaneity or reflection [24,29,31] as underlying built-environment factors or the fact that geographic clustering of people with similar characteristics show a spatial correlation with PV clusters. In this case, we complemented the spatial analysis with a survey. However, it is crucial to find appropriate methods to address these issues when geospatial studies are not supported by additional self-reported data.

In a context like Switzerland, which has varying degrees of urbanisation in a small territory, a considerable presence of physical geography and high relevance of administrative and linguistic boundaries, using straight distance may not reflect the irregularities of the underlying territory, particularly when we look at populations that are already concentrated in the area and at such a fine scale. These observations could play a role in the low significance of the distance factor in the creation of links between adopters and potential adopters when they are calculated as a straight line through the ERGM algorithm. Contextual factors, such as density and uses, or territorial distribution may be more explanatory variables that should be incorporated when proximity is analysed for the formation of referenced links. Additionally, Baranzini [30] and Carattini [59], in a study that specifically looks at the spatial diffusion patterns of PV in Switzerland, have found distance to still be a relevant factor even when contextual variables are controlled for. This indicates that, even if straight distance does not seem to be a significantly explanatory of link creation in the social network, it is still relevant to understand spatial diffusion patterns.

All in all, these observations support the assumption that the organisation of the territory can highly influence the diffusion of PV by (i) creating contextual factors that increment the potential for PV installations and (ii) increasing proximity between adopters and potential adopters and, therefore, accelerating the diffusion. This indicates that more advanced proximity measures are needed when addressing the issues of understanding peer effects dynamics in more depth in the diffusion of energy technologies.

### 4.4 Policy recommendations

The characteristics of the information network and the confirmed importance of spatial proximity in the process of investing in PV installations entails a number of important lessons for future promotion activities, whether they are private or public.

First, neighbourhood effects play an important role when it comes to PV installations. The fact that a neighbour has put up a PV panel significantly influences the probability to put up one oneself. Are important points of reference when it comes to the decision to invest in photovoltaic cells. Second, regional networks are of great importance to actively spread the information in the context of photovoltaic installations. In fact, most of the information exchange takes place within a restricted geographical area of reference, which should facilitate targeted policy initiatives on a regional level which consider regional specificities, such as socio-cultural characteristics as well as regional sensitivities. Third, most people turn to people they know around their centre of life to obtain information regarding the installation of photovoltaic panels. There seems to be considerable untapped potential to use these connections to actively promote PV installations, for example by working with neighbourhood associations or existing structures in other domains, such as the sports and culture sphere, to pass on the message about what can be done and where information can be found. This would allow them to target new people with products and offers in the PV domain who are not naturally interested in the topic but are open to information from their peers, family or friends.

Given that most PV installations are still done by homeowners and that the majority of both information exchangers and referenced people in the network live in the urban areas of the two districts we have looked into leads to the call for a better integration of both tenants and rural populations in the quest for a higher adoption of PV to cover energy need with renewable energy sources in Switzerland, and beyond.

Overall, it could be interesting to use local and regional ambassadors to target groups that have not yet been convinced about investing in photovoltaics. This would help to achieve the carbon emission reductions in electricity production foreseen in the context of the Paris agreement and the Swiss energy strategy 2050 [7], amongst others. In addition, using formal and informal knowledge exchange networks that exist on regional and local scale to set up more decentralised information platforms – both online in the form of targeted websites and social media groups and offline in terms of discussion groups and physical information platforms – would allow a large portion of the population to easily access up-to-date information regarding PV installation in their immediate living environment.

## 5 Conclusion

With the present paper, we aimed to examine determining factors for individuals to install photovoltaic panels in the Canton of Vaud, Switzerland. Specifically, we analysed how geographic properties of knowledge exchange and the role of specific variables, such as income, household composition, the relationship with neighbours and the perception of environmental issues influence the decision to put up PV panels.

Neighbours and knowledge are important and often overlooked drivers of the energy transition. Based on the results of the survey in the canton of Vaud (Switzerland), living next to people who have installed PV panels and being in exchanging with people who have done so significantly enhances the probability of investing in this technology oneself.

When we look at the geographical distribution of the knowledge exchange networks in the field of PV, we can see that the majority of information exchange take place within the linguistic and cultural boundaries of the canton. When the role of physical proximity between actors is analysed, we can see that within given administrative borders, straight distance is not a predictor of knowledge exchange. We suggest that in a complex geographical setting with varying degrees of urbanisation in a small territory, straight distance may not sufficiently reflect the irregularities of the underlying territory. More informed geographical indicators considering cultural, linguistic and administrative factors should be applied.

The study points to three levers to advance the energy transition and shift the system to a predominantly renewable system. (i) First, information exchange between acquaintances should be better considered and better exploited when it comes to initiating and supporting the energy transition. Specifically, political actors, administrations and energy system operators should consider non-professional information exchange between citizens and provide these with information they can share with their network. (ii) Secondly, information campaigns should consider the role of neighbourhood effects when it comes to the installation of solar panels. Ideally, people who have already installed PV panels are involved in such campaigns as ambassadors, reaching out directly to their neighbours and taking on the role of models for their respective communities. (iii) Thirdly, the installation of strong information platforms could counteract the lack of coordination regarding the dissemination of information, considering the advantages of modern communication technology in general, and social networks more specifically.

In terms of future research, it would be interesting to delve deeper into the role of the geographic context that affects proximity effects. On the one hand, it would be useful to develop more comprehensive approaches to measure distance that also consider underlying characteristics of the territory, such as population density, degree of urbanisation or administrative/linguistic boundaries. On the other hand, a deeper understanding of the role of geographic factors when social networks are shaped would be key to obtain more insights about these results and to create effective policies that support information circulation that aids in the diffusion of the technology.

## Author contribution statement

Gloria Serra-Coch: Conceived and designed the experiments; performed the experiments; analysed and interpreted the data and wrote the paper.

Romano Wyss: Conceived and designed the experiments; performed the experiments; analysed and interpreted the data; contributed reagents, materials, analysis tools or data and wrote the paper.

Claudia R. Binder: Conceived and designed the experiments; analysed and interpreted the data and wrote the paper

## Declaration of competing interest

The authors declare that they have no known competing financial interests or personal relationships that could have appeared to influence the work reported in this paper
