# Corrigendum to “Geographic network effects to engage people in the energy transition: The case of PV in Switzerland” [Heliyon 9(7) (July 2023) e17800]

**DOI:** 10.1016/j.heliyon.2023.e19884

**Published:** 2023-09-07

**Authors:** Gloria Serra-Coch, Romano Wyss, Claudia R. Binder

**Affiliations:** a Laboratory for Human Environment Relations in Urban Systems (HERUS), Institute of Environmental Engineering (IIE), School of Architecture, Civil and Environmental Engineering (ENAC), École Polytechnique Fédérale de Lausanne (EPFL), CH-1015, Lausanne, Switzerland; b Wyss Conseil Scientifique, Rue du Bourg 8, CH-1095, Lutry, Switzerland

In the original published version of this article, the percentage in section 3.2 of ‘0.05%’ should be ‘0.5%’. This has been fixed in the correction. The authors apologize for the errors. Both the HTML and PDF versions of the article have been updated to correct the errors

## Declaration of competing interest

The authors declare no conflict of interest.

