# Supplementary material for: Geographic network effects to engage people in the energy transition: The case of PV in Switzerland
Source: Heliyon. 2023 Jun 30;9(7):e17800. doi: 10.1016/j.heliyon.2023.e17800 (PMC10339026; doi:10.1016/j.heliyon.2023.e17800)
Supplement: Multimedia component 1 [file mmc1.docx]

A1: Logit regression on the probability to set up a photovoltaic installation

| **Unweighted cases** |  | **N** | **Percent** |
| --- | --- | --- | --- |
| Selected cases :  Unselected cases  Total | Included in Analysis  Missing Cases  Total | 1125  0  1125  0  1125 | 100.0  .0  100.0  .0  100.0 |

Block 0: Beginning block

Dependent variable encoding

| **Original value** | **Internal value** |
| --- | --- |
| 1  2 | 0  1 |

Classification Table

|  | | | Predicted | | |
| --- | --- | --- | --- | --- | --- |
|  |  |  | pvadopt | | Percentage Correct |
|  | Observed | | 1 | 2 |  |
| Step 0 | pvadopt | 1  2  Global | 0  0 | 413  712 | 0  100  63 |

Variables in the equation

|  | | B | S.E. | Wald | df | Sig | Exp(B) |
| --- | --- | --- | --- | --- | --- | --- | --- |
| Step 0 | Constant | .545 | .062 | 77.532 | 1 | <.001 | 1.724 |

Block 1: introduction

Omnibus Test of Model Coefficients

|  | | Chi-square | df | Sig. |
| --- | --- | --- | --- | --- |
| Step 1 | Step  Block  Model | 158.358  158.358  158.358 | 28  28  28 | <.001  <.001  <.001 |

Model summary:

| Step | -2 Log Likehood | Cox & Snell R square | Nagelkerke R Square |
| --- | --- | --- | --- |
| 1 | 1320.792^a^ | .131 | .180 |

Classification Table^a^

|  | | | Predicted | | |
| --- | --- | --- | --- | --- | --- |
|  |  |  | C07 | | Percentage Correct |
|  | Observed | | 1 | 2 |  |
| Step 1 | C07 | 1  2  Global | 183  144 | 230  568 | 44.3  79.8  66.8 |

a: the cut value is .500

Variables in the Equation

| **Explanatory variables** | **B** | **S.E.** | **Wald** | **df** | **Sig.** | **Exp (B)** |
| --- | --- | --- | --- | --- | --- | --- |
| Age (A01AGE) | ,008 | ,006 | 1,994 | 1 | ,158 | 1,008 |
| Sex (A02) | ,030 | ,069 | ,188 | 1 | ,665 | 1,031 |
| Household composition 0-17 (A03_1) | -,071 | ,077 | ,855 | 1 | ,355 | ,931 |
| Household composition 18-35 (A03_2) | ,032 | ,057 | ,320 | 1 | ,571 | 1,033 |
| Household composition 36-50 (A03_3) | -,006 | ,023 | ,078 | 1 | ,779 | ,994 |
| Household composition 51-65 (A03_4) | ,004 | ,011 | ,137 | 1 | ,712 | 1,004 |
| Household composition 66+ (A03_5) | -,006 | ,006 | 1,017 | 1 | ,313 | ,994 |
| **Home Ownership (A04)** | **1,312** | **,196** | **44,757** | **1** | **<,001** | **3,712***** |
| Type of dwelling (A05) | -,005 | ,007 | ,505 | 1 | ,477 | ,995 |
| Ownership duration (A06) | ,016 | ,028 | ,319 | 1 | ,572 | 1,016 |
| Relationship with the neighbours (A07) | ,001 | ,008 | ,030 | 1 | ,861 | 1,001 |
| Identification with the neighbourhood (A08) | ,005 | ,003 | 2,334 | 1 | ,127 | 1,005 |
| Responsibility of energy expenditure (A09) | -,002 | ,082 | ,000 | 1 | ,984 | ,998 |
| Interest in the environment (A10) | ,006 | ,006 | ,786 | 1 | ,375 | 1,006 |
| Exchanges about environmental questions (A11) | ,004 | ,015 | ,076 | 1 | ,783 | 1,004 |
| **Panel installation by people you know (C09)** | **,637** | **,139** | **21,001** | **1** | **<,001** | **1,891***** |
| **Installation of PV panels by neighbours (C14)** | **,005** | **,002** | **5,420** | **1** | **,020** | **1,005*** |
| Exchange with neighbours (C14) | ,003 | ,005 | ,318 | 1 | ,573 | 1,003 |
| Advice given in the past (C15) | -,204 | ,168 | 1,471 | 1 | ,225 | ,815 |
| Consideration of own impact on environment (E01_1) | -,004 | ,006 | ,462 | 1 | ,497 | ,996 |
| Impact of habits on environment (E01_2) | ,004 | ,007 | ,296 | 1 | ,586 | 1,004 |
| Preoccupation with climate impact of energy use (E01_3) | ,015 | ,009 | 3,183 | 1 | ,074 | 1,015 |
| Support of environmental protection (E01_4) | -,011 | ,009 | 1,418 | 1 | ,234 | ,989 |
| Priority given to environmental issues (E01_5) | ,003 | ,006 | ,294 | 1 | ,588 | 1,003 |
| **Professional status (E02)** | **-,010** | **,004** | **5,173** | **1** | **,023** | **,990*** |
| Civil status (E03) | ,007 | ,008 | ,673 | 1 | ,412 | 1,007 |
| Level of revenue (E04) | ,001 | ,002 | ,091 | 1 | ,763 | 1,001 |
| Level of education (E05) | ,000 | ,007 | ,001 | 1 | ,977 | 1,000 |
| Constant | -2,530 | ,594 | 18,138 | 1 | <,001 | ,080 |

A2: Correlation Matrix and Variance Inflation Factor (VIF)

|  | Const. | A01 | A02 | A03_1 | A03_2 | A03_3 | A03_4 | A03_5 | A04 | A05 | A06 | A07 | A08 | A09 | A10 | A11 | C09 | C13 | C14 | C15 | E01_1 | E01_2 | E01_3 | E01_4 | E01_5 | E02 | E03 | E04 | E05 |
| --- | --- | --- | --- | --- | --- | --- | --- | --- | --- | --- | --- | --- | --- | --- | --- | --- | --- | --- | --- | --- | --- | --- | --- | --- | --- | --- | --- | --- | --- |
| Const. | 1,000 | -,639 | -,153 | -,217 | ,006 | ,055 | -,028 | ,114 | -,459 | -,027 | -,246 | -,015 | -,036 | -,238 | -,014 | -,058 | -,256 | ,026 | ,084 | -,414 | ,016 | ,019 | ,005 | ,039 | -,047 | ,024 | -,008 | ,004 | -,082 |
| A01 | -,639 | 1,000 | -,070 | ,245 | ,038 | -,031 | ,024 | -,206 | ,145 | -,032 | -,178 | ,031 | ,048 | ,117 | ,063 | ,023 | ,016 | ,025 | -,092 | ,027 | -,021 | -,018 | ,008 | -,018 | ,032 | -,005 | ,021 | -,015 | -,002 |
| A02 | -,153 | -,070 | 1,000 | ,000 | -,004 | -,008 | ,002 | ,008 | -,043 | -,004 | -,089 | ,033 | ,011 | ,013 | -,019 | -,043 | ,026 | ,032 | -,043 | ,030 | -,004 | ,034 | ,036 | -,076 | ,021 | ,027 | -,020 | ,056 | -,053 |
| A03_1 | -,217 | ,245 | ,000 | 1,000 | -,455 | -,339 | ,057 | -,015 | ,093 | ,029 | ,058 | ,029 | ,047 | -,025 | ,018 | ,007 | ,014 | ,013 | -,088 | ,012 | ,008 | -,010 | -,004 | -,002 | -,026 | ,018 | ,029 | ,046 | -,012 |
| A03_2 | ,006 | ,038 | -,004 | -,455 | 1,000 | -,208 | -,011 | ,021 | -,040 | -,034 | -,067 | ,005 | -,004 | -,053 | -,022 | -,003 | -,003 | ,027 | -,086 | ,006 | -,014 | -,012 | -,014 | ,012 | ,018 | ,030 | ,014 | -,015 | -,014 |
| A03_3 | ,055 | -,031 | -,008 | -,339 | -,208 | 1,000 | ,008 | ,005 | -,051 | ,004 | ,007 | ,019 | -,042 | ,003 | ,023 | ,004 | -,043 | ,006 | -,018 | -,001 | ,021 | ,004 | -,003 | -,006 | ,024 | -,049 | -,008 | ,014 | ,019 |
| A03_4 | -,028 | ,024 | ,002 | ,057 | -,011 | ,008 | 1,000 | ,036 | ,026 | ,009 | -,004 | ,008 | ,021 | -,021 | ,011 | ,013 | ,019 | ,015 | -,030 | -,055 | ,030 | -,076 | ,022 | ,011 | ,009 | ,026 | ,023 | -,024 | -,005 |
| A03_5 | ,114 | -,206 | ,008 | -,015 | ,021 | ,005 | ,036 | 1,000 | -,040 | -,047 | ,008 | ,024 | -,039 | -,008 | ,017 | -,008 | -,023 | ,011 | -,037 | ,001 | ,021 | ,033 | -,043 | ,033 | -,048 | ,007 | -,002 | ,030 | ,024 |
| A04 | -,459 | ,145 | -,043 | ,093 | -,040 | -,051 | ,026 | -,040 | 1,000 | ,117 | ,479 | -,018 | -,040 | -,162 | -,014 | ,039 | -,010 | -,080 | -,011 | -,088 | -,027 | -,021 | -,025 | ,015 | -,002 | -,080 | ,000 | -,022 | ,074 |
| A05 | -,027 | -,032 | -,004 | ,029 | -,034 | ,004 | ,009 | -,047 | ,117 | 1,000 | ,001 | ,017 | -,029 | -,014 | -,089 | -,007 | ,002 | -,017 | ,025 | -,048 | ,004 | -,003 | ,008 | ,004 | ,003 | -,059 | ,004 | ,021 | ,016 |
| A06 | -,246 | -,178 | -,089 | ,058 | -,067 | ,007 | -,004 | ,008 | ,479 | ,001 | 1,000 | ,000 | -,013 | ,045 | -,015 | ,015 | ,007 | ,032 | -,006 | ,029 | ,005 | ,025 | -,077 | ,019 | ,024 | -,025 | ,024 | -,069 | ,021 |
| A07 | -,015 | ,031 | ,033 | ,029 | ,005 | ,019 | ,008 | ,024 | -,018 | ,017 | ,000 | 1,000 | -,081 | ,013 | ,000 | -,231 | ,006 | ,006 | -,240 | -,032 | -,042 | ,067 | -,029 | -,023 | ,042 | -,030 | -,036 | ,013 | -,049 |
| A08 | -,036 | ,048 | ,011 | ,047 | -,004 | -,042 | ,021 | -,039 | -,040 | -,029 | -,013 | -,081 | 1,000 | ,030 | -,020 | -,002 | ,009 | -,004 | -,025 | -,025 | ,010 | ,054 | ,013 | -,040 | -,087 | -,034 | ,050 | -,026 | -,079 |
| A09 | -,238 | ,117 | ,013 | -,025 | -,053 | ,003 | -,021 | -,008 | -,162 | -,014 | ,045 | ,013 | ,030 | 1,000 | -,023 | ,023 | -,027 | -,058 | -,019 | -,034 | -,059 | ,053 | ,028 | -,062 | ,023 | -,012 | -,020 | ,025 | ,020 |
| A10 | -,014 | ,063 | -,019 | ,018 | -,022 | ,023 | ,011 | ,017 | -,014 | -,089 | -,015 | ,000 | -,020 | -,023 | 1,000 | -,011 | -,043 | ,024 | -,061 | -,016 | ,027 | ,001 | -,014 | -,017 | -,027 | -,011 | ,034 | -,003 | -,023 |
| A11 | -,058 | ,023 | -,043 | ,007 | -,003 | ,004 | ,013 | -,008 | ,039 | -,007 | ,015 | -,231 | -,002 | ,023 | -,011 | 1,000 | -,040 | -,013 | -,083 | -,025 | -,010 | -,012 | -,010 | -,027 | ,020 | -,101 | ,067 | -,073 | ,045 |
| C09 | -,256 | ,016 | ,026 | ,014 | -,003 | -,043 | ,019 | -,023 | -,010 | ,002 | ,007 | ,006 | ,009 | -,027 | -,043 | -,040 | 1,000 | -,131 | -,022 | -,176 | -,002 | -,037 | ,033 | -,016 | -,004 | -,010 | ,009 | -,076 | -,027 |
| C13 | ,026 | ,025 | ,032 | ,013 | ,027 | ,006 | ,015 | ,011 | -,080 | -,017 | ,032 | ,006 | -,004 | -,058 | ,024 | -,013 | -,131 | 1,000 | -,077 | ,005 | -,007 | ,010 | -,025 | ,009 | -,016 | -,004 | ,044 | -,010 | -,014 |
| C14 | ,084 | -,092 | -,043 | -,088 | -,086 | -,018 | -,030 | -,037 | -,011 | ,025 | -,006 | -,240 | -,025 | -,019 | -,061 | -,083 | -,022 | -,077 | 1,000 | -,029 | ,017 | -,007 | ,019 | ,078 | -,115 | -,012 | -,077 | -,110 | ,042 |
| C15 | -,414 | ,027 | ,030 | ,012 | ,006 | -,001 | -,055 | ,001 | -,088 | -,048 | ,029 | -,032 | -,025 | -,034 | -,016 | -,025 | -,176 | ,005 | -,029 | 1,000 | ,021 | -,035 | -,027 | -,021 | ,043 | ,009 | ,000 | ,003 | ,022 |
| E01_1 | ,016 | -,021 | -,004 | ,008 | -,014 | ,021 | ,030 | ,021 | -,027 | ,004 | ,005 | -,042 | ,010 | -,059 | ,027 | -,010 | -,002 | -,007 | ,017 | ,021 | 1,000 | -,233 | -,122 | -,148 | -,178 | -,025 | ,064 | -,068 | -,016 |
| E01_2 | ,019 | -,018 | ,034 | -,010 | -,012 | ,004 | -,076 | ,033 | -,021 | -,003 | ,025 | ,067 | ,054 | ,053 | ,001 | -,012 | -,037 | ,010 | -,007 | -,035 | -,233 | 1,000 | -,246 | -,148 | -,127 | -,013 | -,147 | -,012 | ,024 |
| E01_3 | ,005 | ,008 | ,036 | -,004 | -,014 | -,003 | ,022 | -,043 | -,025 | ,008 | -,077 | -,029 | ,013 | ,028 | -,014 | -,010 | ,033 | -,025 | ,019 | -,027 | -,122 | -,246 | 1,000 | -,488 | -,168 | ,030 | ,076 | ,034 | -,096 |
| E01_4 | ,039 | -,018 | -,076 | -,002 | ,012 | -,006 | ,011 | ,033 | ,015 | ,004 | ,019 | -,023 | -,040 | -,062 | -,017 | -,027 | -,016 | ,009 | ,078 | -,021 | -,148 | -,148 | -,488 | 1,000 | -,227 | -,005 | -,091 | -,052 | -,045 |
| E01_5 | -,047 | ,032 | ,021 | -,026 | ,018 | ,024 | ,009 | -,048 | -,002 | ,003 | ,024 | ,042 | -,087 | ,023 | -,027 | ,020 | -,004 | -,016 | -,115 | ,043 | -,178 | -,127 | -,168 | -,227 | 1,000 | -,038 | -,071 | -,010 | -,036 |
| E02 | ,024 | -,005 | ,027 | ,018 | ,030 | -,049 | ,026 | ,007 | -,080 | -,059 | -,025 | -,030 | -,034 | -,012 | -,011 | -,101 | -,010 | -,004 | -,012 | ,009 | -,025 | -,013 | ,030 | -,005 | -,038 | 1,000 | -,246 | -,032 | -,062 |
| E03 | -,008 | ,021 | -,020 | ,029 | ,014 | -,008 | ,023 | -,002 | ,000 | ,004 | ,024 | -,036 | ,050 | -,020 | ,034 | ,067 | ,009 | ,044 | -,077 | ,000 | ,064 | -,147 | ,076 | -,091 | -,071 | -,246 | 1,000 | -,108 | -,221 |
| E04 | ,004 | -,015 | ,056 | ,046 | -,015 | ,014 | -,024 | ,030 | -,022 | ,021 | -,069 | ,013 | -,026 | ,025 | -,003 | -,073 | -,076 | -,010 | -,110 | ,003 | -,068 | -,012 | ,034 | -,052 | -,010 | -,032 | -,108 | 1,000 | -,048 |
| E05 | -,082 | -,002 | -,053 | -,012 | -,014 | ,019 | -,005 | ,024 | ,074 | ,016 | ,021 | -,049 | -,079 | ,020 | -,023 | ,045 | -,027 | -,014 | ,042 | ,022 | -,016 | ,024 | -,096 | -,045 | -,036 | -,062 | -,221 | -,048 | 1,000 |

| Variable | VIF | Variable | VIF | Variable | VIF |
| --- | --- | --- | --- | --- | --- |
| A01AGE 1.471381  A02 1.346571  A03_1 1.939689  A03_2 1.538432  A03_3 1.440770  A03_4 1.022859  A03_5 1.057198  A04 1.247636  A05 1.106062  A06 1.178594  A07 1.273408  A08 1.106415 | | A09 1.139006  A10 1.151033  A11 1.381861  C09 1.111969  C14 1.343582  C15 1.074190  E01_1 2.235866  E01_2 2.879516  E01_3 3.705597  E01_4 2.971020  E01_5 3.054822  E02 1.240685 | | E03 1.531358  E04 1.178405  E05 1.412147 | |

A3: Results of the ERGM model.

|  |  | Estimate | Std. Error | MCMC% | Z value | PR (>\|z\|) |
| --- | --- | --- | --- | --- | --- | --- |
| Link - Postcode | 1180  1273  1400 | 1.51037  2.04318  0.59030 | 0.70908  0.71016  0.15291 | 0  0  0 | 2.130  2.877  3.860 | 0.033167 *  0.004014 **  0.000113 *** |
| Link - Municipality | **Arzier-Le Muids**  Chavornay  Gland  Grandson  Le Sentier  Nyon  Orbe  Prangings  **Rolle**  Ste-Croix  **Yverdon-les-Bains**  Yvonand | 2.04228  0.0508  0.25621  0.80767  0.32083  -0.03624  -0.16233  0.96698  1.50947  -0.68727  0.53882  0.50342 | 0.71015  0.70790  0.44817  0.70830  1.00097  0.40919  0.70783  0.57861  0.70907  1.00055  0.17864  0.70810 | 0  0  0  0  0  0  0  0  0  0  0  0 | 2.876  0.072  0.572  1.140  0.321  -0.089  -0.229  1.671  2.129  -0.687  3.016  0.711 | 0.00403 **  0.94273  0.56754  0.25417  0.74857  0.92943  0.81861  0.09468 .  0.03327 *  0.49215  0.00256 **  .47712 |
| Link - Distance |  | -7.238e-08 | 1.032e-07 | 0 | -0.701 | 0.483 |

Significance levels: *** = 0 ** = 0.001 *= 0.05

A4: Average Nearest Neighbor Summary

| **Average Nearest Neighbor Results** | **Respondents + Referenced people** | **Respondents** | **Referenced people** | **PV installations in Vaud** |
| --- | --- | --- | --- | --- |
| **Observed Mean Distance** | 4369.3347m | 47.4273m | 11438.7552m | 75.0430m |
| **Expected Mean Distance** | 41540.9968m | 3590.2575 | 67193.5005 | 414.6891 |
| **Nearest Neighbor Ratio** | 0.104965 | 0.013210 | 0.170236 | 0.180962 |
| **z-score** | -73.067805 | -63.318685 | -41.878399 | -158.665148 |
| **p-value** | 0.000 | 0.000 | 0.000 | 0.000 |

A5: Results of the geographic analysis

**
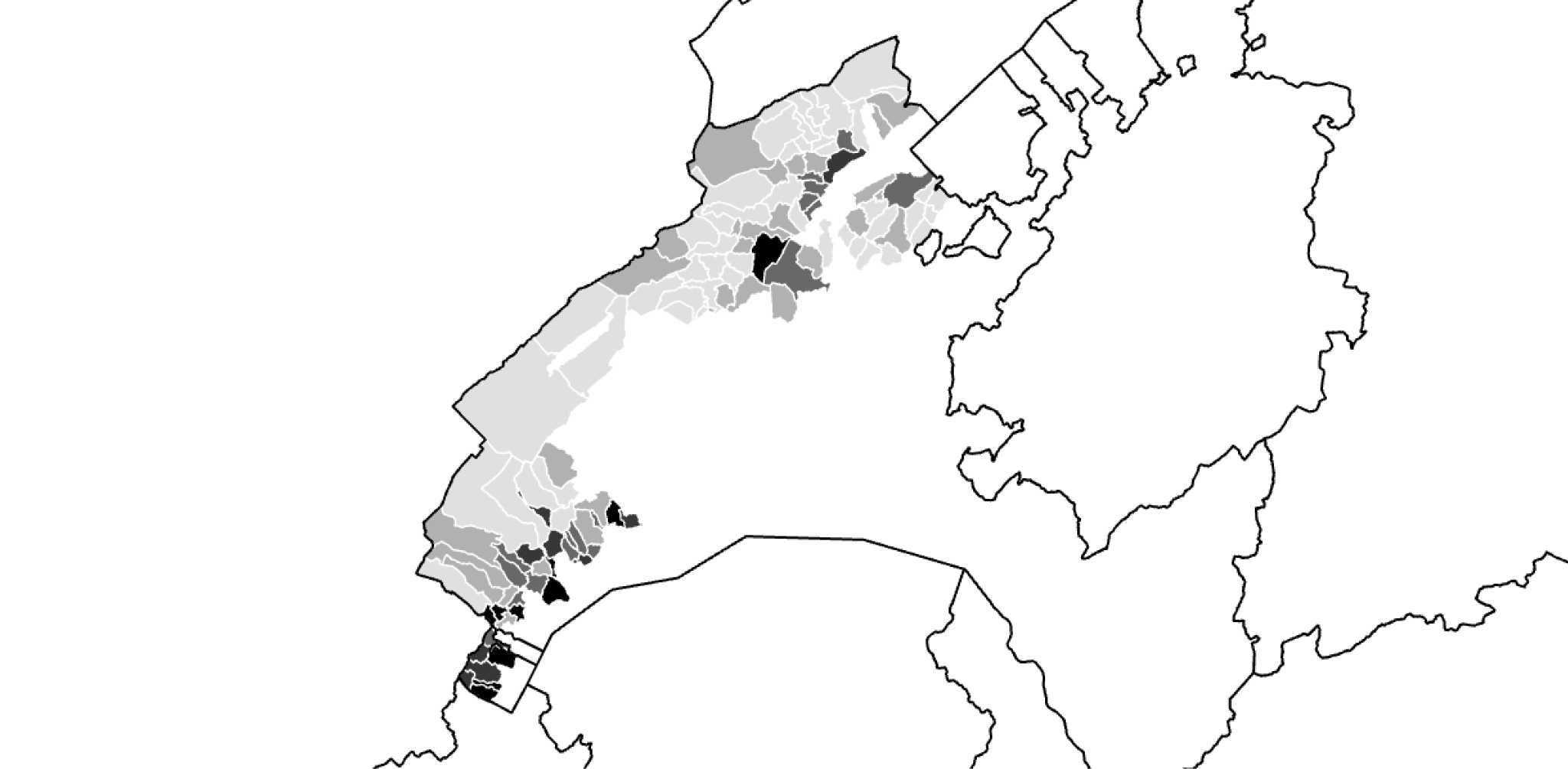

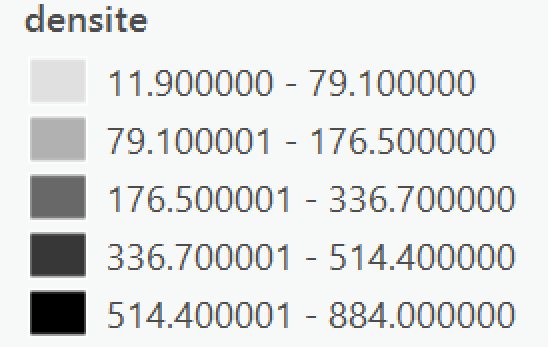
**

Figure A5-1: Population density in case study region


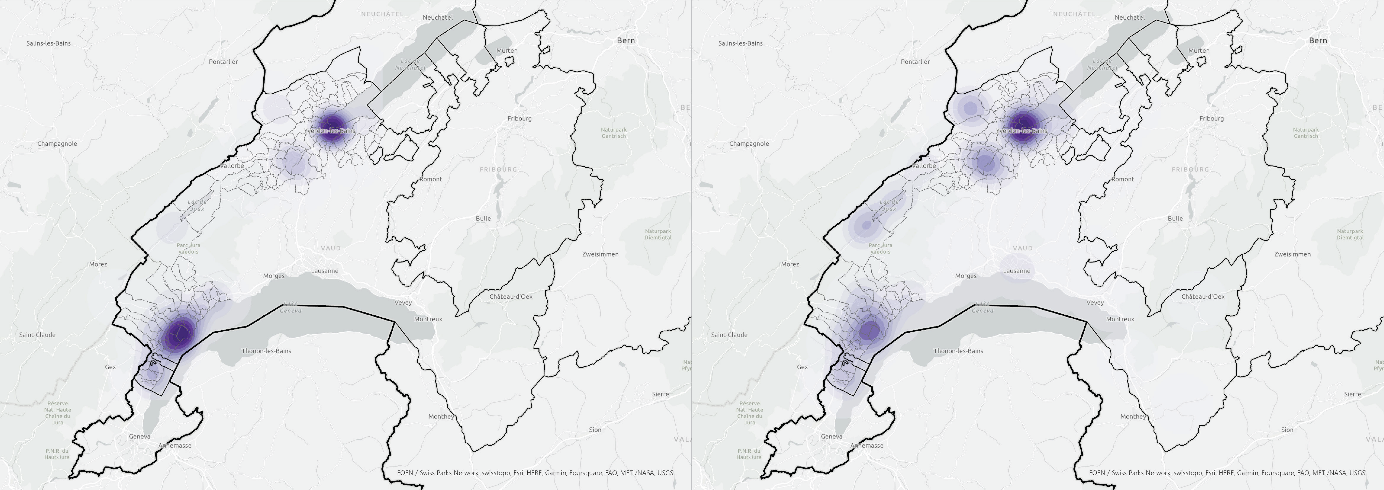


Figure A5-2: Kernel density map of information exchangers (left) and referenced (right)


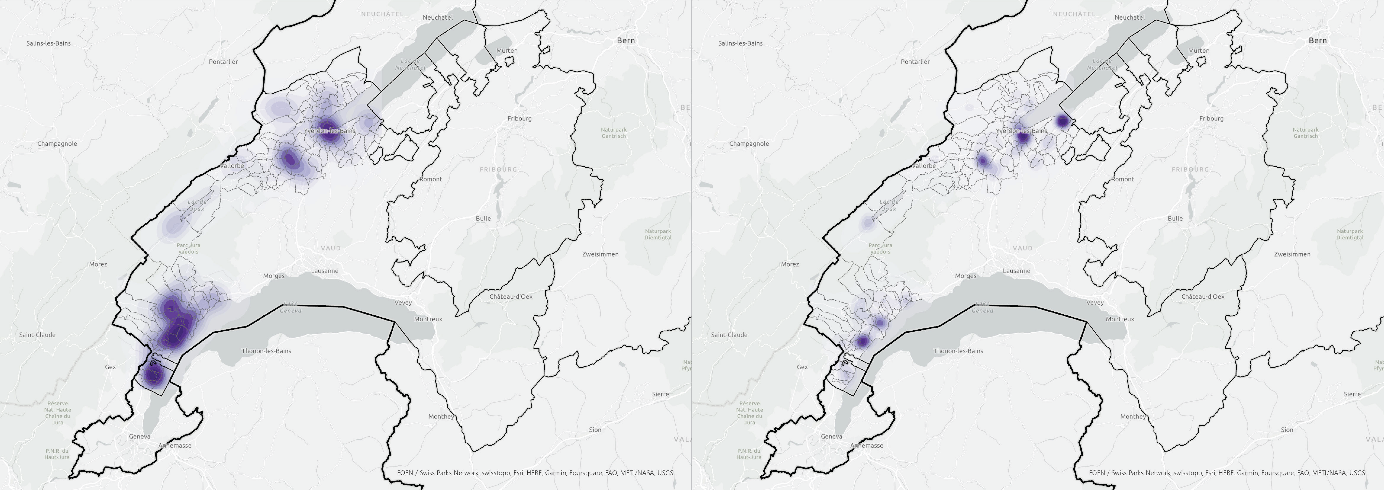


Figure A5-3: Kernel density map of PV installations (left) and PV power (right)


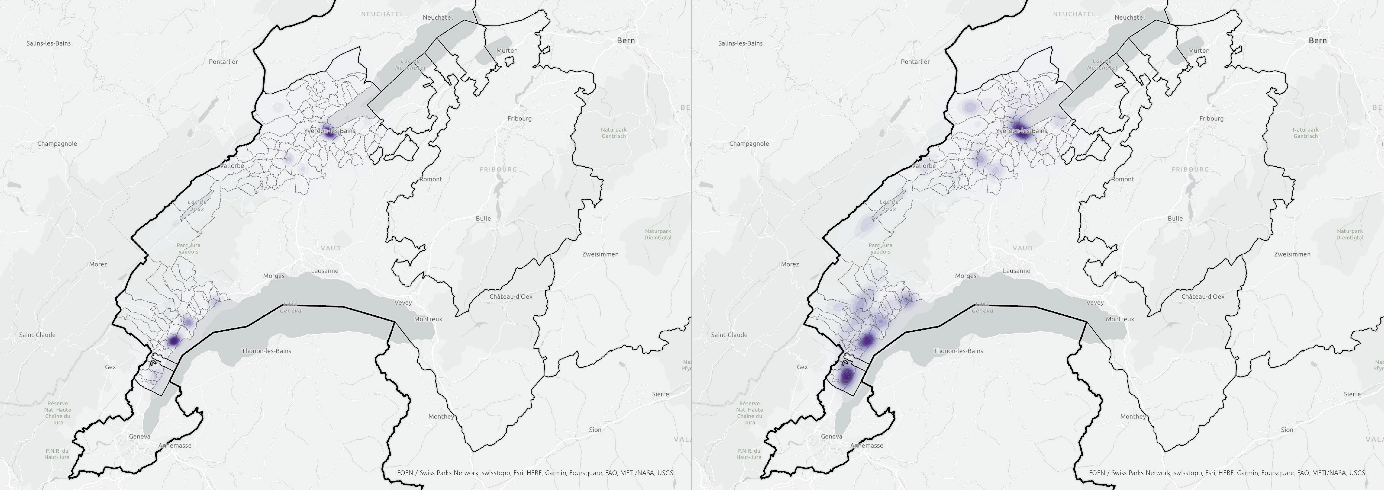


Figure A5-4: Kernel density map of population (left) and housing units (right)


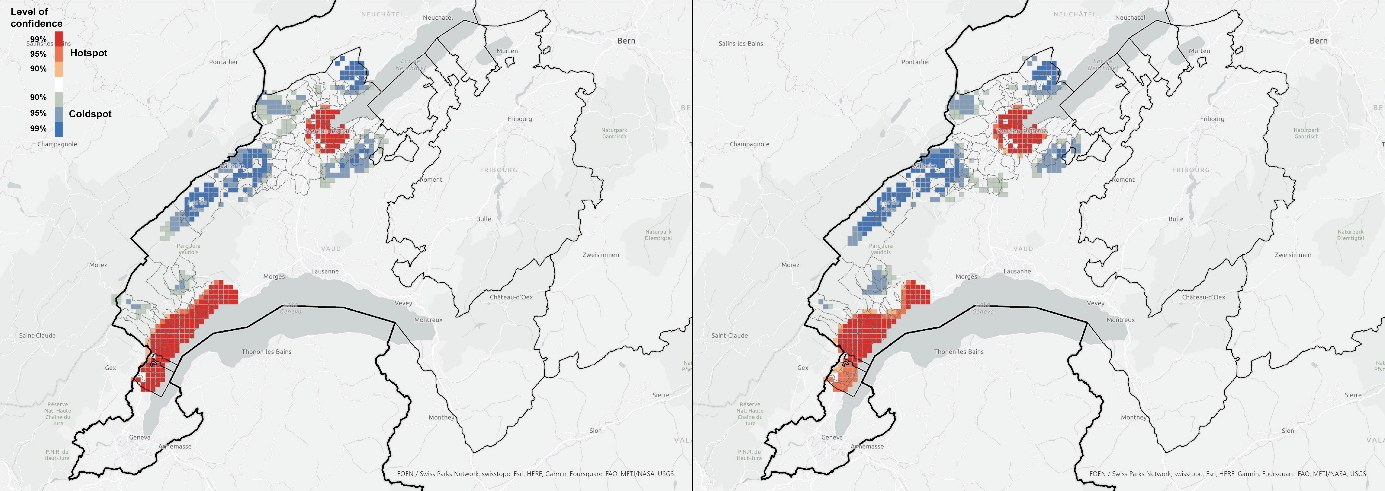


Figure A5-5: Hotspot analysis in a fishnet grid of 1-km cells at the case study level of neighborhood services accessibility (left) food services accessibility (right)

**
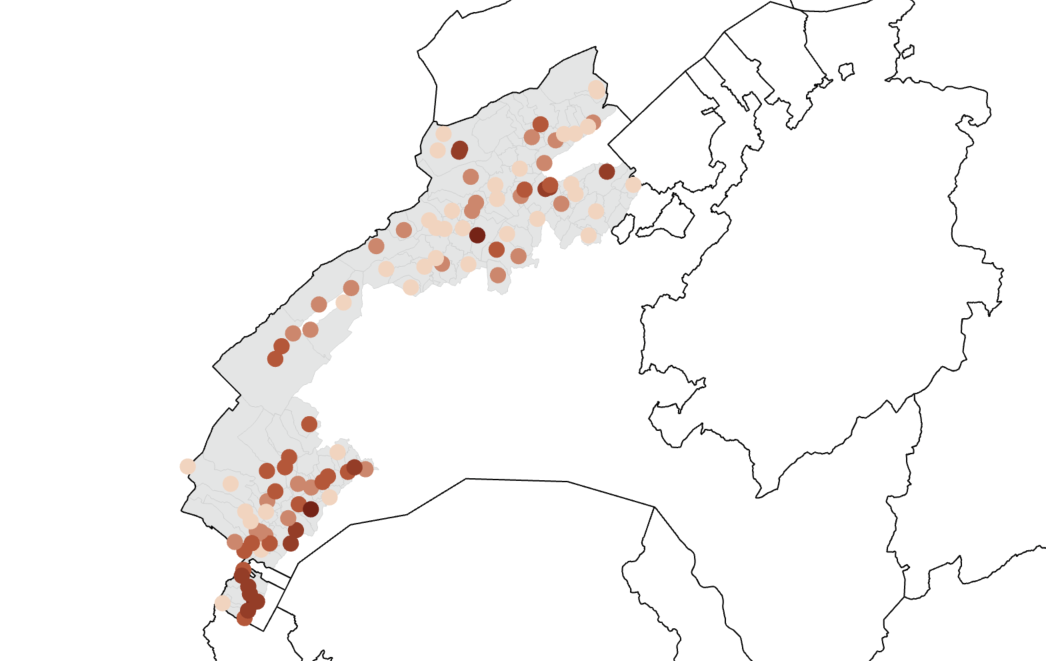
** **
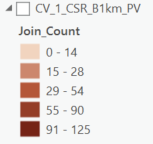
** **
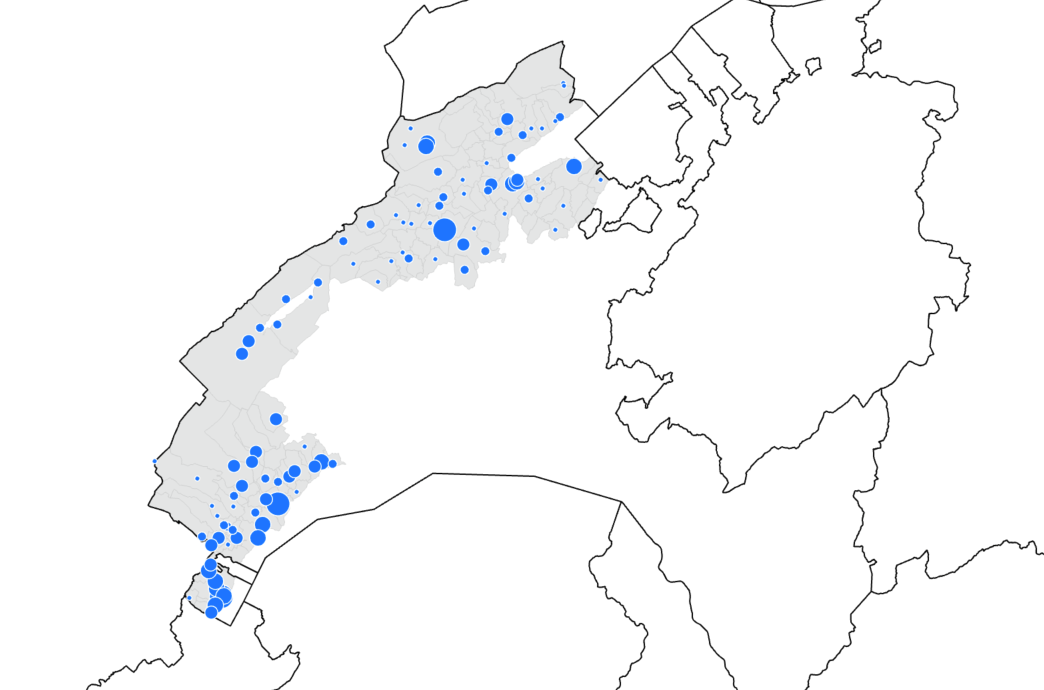
** **
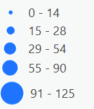
**

Figure A5-6: Respondents and the number PV installations within 1km buffer around them.

**
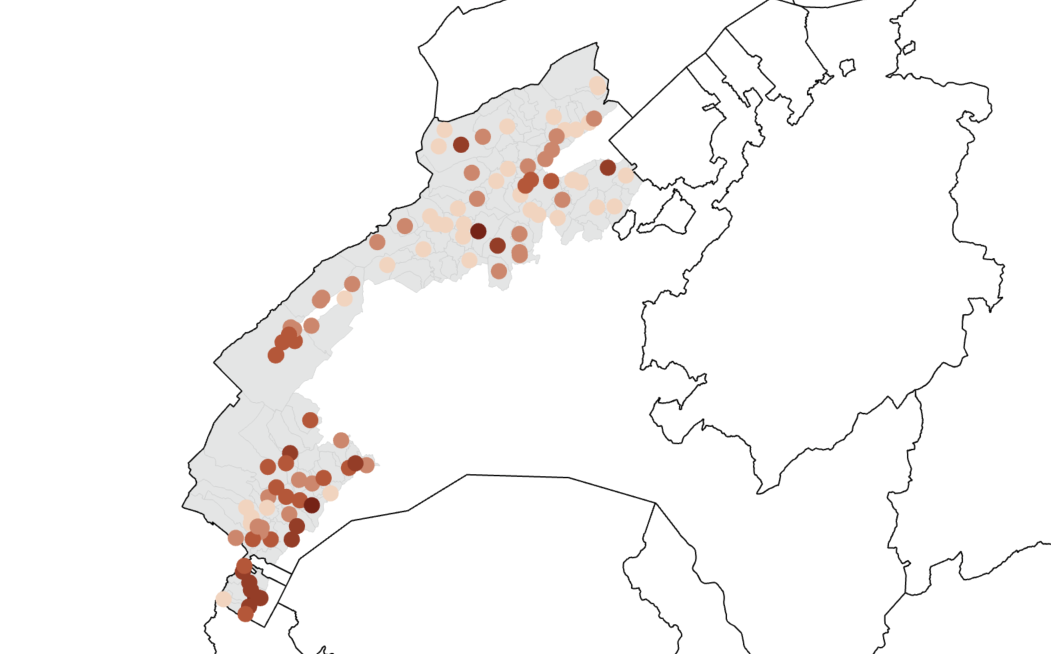
** **
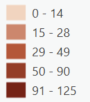
** **
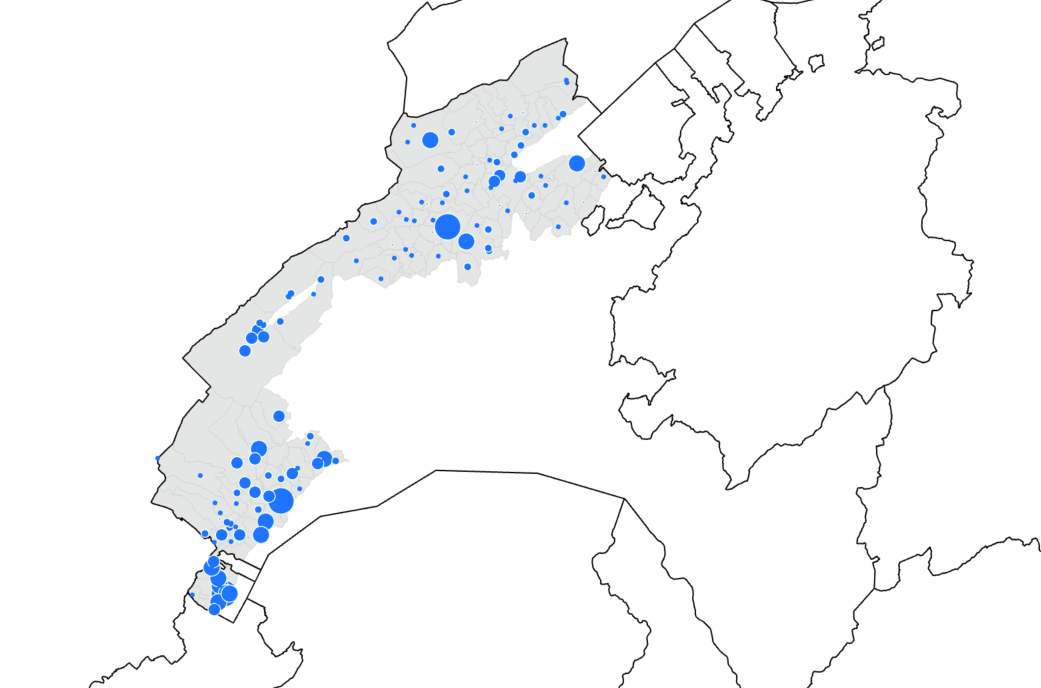
** **
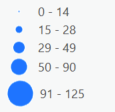
**

Figure A5-7: Referenced people and the number PV installations within 1km buffer around them within the case study area.
